# Clarifying assumptions in age-period-cohort analyses and validating results

**DOI:** 10.1371/journal.pone.0238871

**Published:** 2020-10-06

**Authors:** Ryan Masters, Daniel Powers

**Affiliations:** 1 University of Colorado Boulder, Boulder, CO, United States of America; 2 University of Texas at Austin, Austin, Texas, United States of America; University of California-Irvine, UNITED STATES

## Abstract

**Background:**

Age-period-cohort (APC) models are often used to decompose health trends into period- and cohort-based sources, but their use in epidemiology and population sciences remains contentious. Central to the contention are researchers’ failures to 1) clearly state their analytic assumptions and/or 2) thoroughly evaluate model results. These failures often produce varying conclusions across APC studies and generate confusion about APC methods. Consequently, scholarly exchanges about APC methods usually result in strong disagreements that rarely offer practical advice to users or readers of APC methods.

**Methods:**

We use research guidelines to help practitioners of APC methods articulate their analytic assumptions and validate their results. To demonstrate the usefulness of the guidelines, we apply them to a 2015 *American Journal of Epidemiology* study about trends in black-white differences in U.S. heart disease mortality.

**Results:**

The application of the guidelines highlights two important findings. On the one hand, some APC methods produce inconsistent results that are highly sensitive to researcher manipulation. On the other hand, other APC methods estimate results that are robust to researcher manipulation and consistent across APC models.

**Conclusions:**

The exercise shows the simplicity and effectiveness of the guidelines in resolving disagreements over APC results. The cautious use of APC models can generate results that are consistent across methods and robust to researcher manipulation. If followed, the guidelines can likely reduce the chance of publishing variable and conflicting results across APC studies.

## Introduction

A 2015 article in the *American Journal of Epidemiology* featured cohort analyses to examine trends in black-white differences in U.S. heart disease mortality rates [1]. Accompanying the article was a comment and a reply by two of the article’s authors that discussed the merits of using age-period-cohort (APC) methods to identify cohort-based trends [2, 3]. The exchange exemplified the contentious spirit that characterizes most discussions about APC methods (e.g., see [4–12]). The back-and-forth was unsatisfactory as the exchange provided little practical advice for users of APC methods. On one side was a group of APC practitioners who enthusiastically supported the use of an “APC toolbox” [3: 1] and on the other side was a skeptic warning of severe “potential pitfalls” in APC analyses [2: 1]. Harper [2] rightly emphasized that the utility of APC models rests on the plausibility of one’s assumptions, but casted doubt on the use of all APC methods. Pointing out that APC models have estimated “wildly differing conclusions regarding the influence of period and cohort effects” on U.S. heart disease mortality, Harper [2: 2] warned, “it seems likely that researchers could end up choosing APC models that are most consistent with their favorite hypotheses.” In response to Harper’s critical assessment of APC methods, Kramer and Casper [3: 1] believed the exchange produced “more areas of agreement than disagreement,” and left with their results largely intact. They concluded, “the APC toolbox accomplished the task for which it is suited.”

Readers of the exchange were left at an impasse, with neither a way to assess the points raised in the exchange nor a clear way forward to use APC models or interpret their results. Should readers distrust all results from APC analyses or does an “APC toolbox” exist for their easy use? We suggest it’s neither of these alternatives and recommend three guidelines for the cautious use of APC methods in social science research. The guidelines build on prior work, and we briefly review how and why the guidelines can provide useful frameworks for APC researchers to follow [10, 13, 14]. We then revisit Kramer et al.’s [1] analyses and apply the guidelines to assess the use of APC methods in light of the exchange between Harper [2] and Kramer and Casper [3]. The exercise illustrates the merits of the guidelines in resolving disagreements over APC results. The exercise provides evidence against the notion that a general “APC toolbox” exists for easy analysis of cohort trends. Some APC methods are preferred to others and researchers need to adjudicate between them when fitting APC models. Indeed, some APC methods estimate results that are *internally consistent* (i.e., results are invariable to model specification) as well as *consistent with other methods* (i.e., results estimated from different APC methods are substantively and statistically nondifferent from each other). In contrast, other APC methods estimate results that are neither internally consistent nor consistent with other methods’ estimates. Yet, findings from our exercise also rebuke Harper’s contention that researchers can manipulate APC estimates to be “most consistent with their favorite hypotheses” [2: 2]. Conclusions differ across APC studies largely because researchers misapply APC methods and fail to validate results, not necessarily because the methods are susceptible to manipulation. What follows is a discussion and application of APC guidelines to first, provide steps to help clarify the use of APC methods in social science research and second, perform sensitivity tests to validate results.

### Guidelines for APC analyses

Cohort analyses have a long history in demographic and epidemiological research, but doubts remain about using APC statistical methods to investigate cohort-based sources of trends [4, 5, 7–9, 11–29]. The first cohort analyses plotted age-specific rates across time periods to descriptively show variation in trends [20]. These practices continue to serve as first steps for identifying “non-parallelism” in age-specific trends, which can indicate cohort-based variation in the outcome [13]. Thus, descriptive plots help researchers decide if they ought to employ a full “three-dimensional” (i.e., APC) statistical model over a simpler age-period (AP) or age-cohort model (AC). However, these descriptive plots do not help determine which statistical approach is preferred for fitting an APC model [13, 14]. This is an important point of clarification that challenges the idea that an “APC toolbox” exists. Although graphical plots might suggest an APC model would be useful for detecting age-, period-, and cohort-based sources of variation in an outcome, researchers must choose from a number of statistically-based APC methods to help investigate their research questions about cohort effects. The following guidelines apply to this choice.

#### Simplify models and explicitly state assumptions

A central concern with fitting statistical APC models is the constraint imposed by a given method [5, 14, 19, 21, 23, 27–33]. A constraint is necessary in order to identify *a* solution from the *infinite* number of solutions that exist as a result of the linear dependency between age, period, and cohort (e.g., cohort = period-age). Clearly stating the assumptions behind a statistical method’s constraint is necessary as some constraints are more appropriate than others for a given data structure [14]. Further, model estimates from some APC methods can vary considerably depending on the choice of constraint [7–10, 21, 30]. Finally, some “explicit constraints” are imposed directly by researchers whereas other methods impose “mechanical constraints” that are less influenced by researchers’ decisions [19, 33].

Kramer et al. [1] took a “coefficients-constraint approach” and specified a Constrained Generalized Linear Model (CGLIM) using dummy-variable coding with explicit equality constraints on the first two time periods [4, 14, 18]. Use of this explicit constraint requires strong theory and/or empirical evidence to assume equality between APC referent categories and the constrained parameter values. In models with highly collinear predictors, dummy-variable designs like this privilege a particular solution in the solution space [13]. It is for this reason that early developers of some APC methods used centered effects coding (i.e., sum to zero constraints over each set of APC parameters) and constrained parameter invariance to small regions in the parameter space [30, 31, 34]. The application of an equality constraint on parameters requires even stronger theory or *a priori* “side information” to justify setting parameter values equal to each other [4, 13, 14, 21: 22, 23, 35].

Kramer et al. [1], for instance, constrained the first two periods in their model to be equal to each other, and used the seventh age and sixth cohort as referent categories: $\beta_{7}^{A}=\beta_{1}^{P}=\beta_{2}^{P}=\beta_{6}^{C}=0$. Moreover, they applied the constraint in a “somewhat unorthodox” way by fitting an APC model that constrained the period parameters associated with the rate *ratio* between black and white mortality, rather than fitting APC models separately to the mortality rates in each population (i.e., separately fitting APC models to black women, white women, black men, and white men) [3: 1]. Although APC models can be fitted to rate ratios, the approach unnecessarily complicates the constraint assumptions and is difficult to theoretically justify or to empirically evaluate. The constraint is both difficult for readers to follow and also increases the chance that model estimates of APC parameters are biased because the imposed $\beta_{7}^{A}=\beta_{1}^{P}=\beta_{2}^{P}=\beta_{6}^{C}$ equality constraint on the rate ratios must hold simultaneously in separate populations. This is because Kramer et al. fitted the APC model to black-white rate ratios in heart disease mortality among a pooled sample of black and white men and women and used interaction terms to estimate gender variability in the APC effects on the rate ratios. The pooled model fitted to rate ratios using interaction terms will estimate APC effects that are statistically indistinguishable from estimates of rate ratios derived from APC models fitted separately to the race/ethnic- and gender-specific populations. Yet, the single model’s estimates and assumptions are difficult for readers to follow and verify.

**Fig 1:**
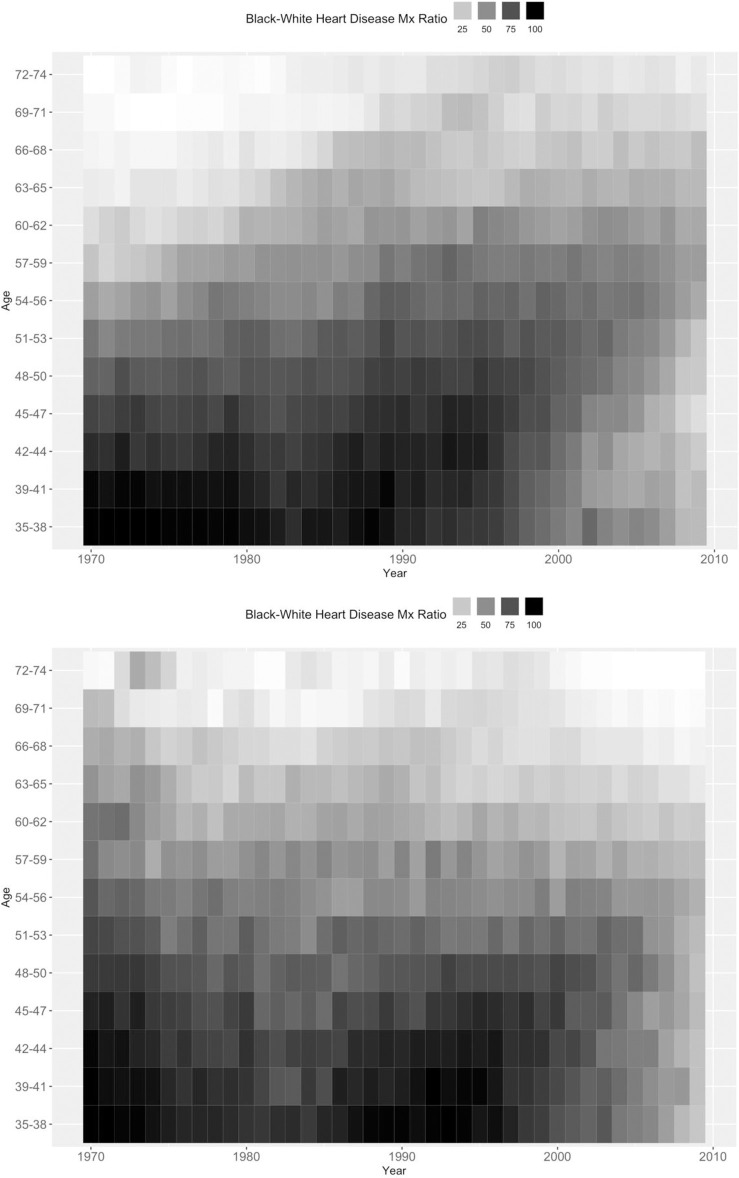
Black-white heart disease mortality rate ratios by age and period. Men on top and women on bottom.

**Fig 2:**
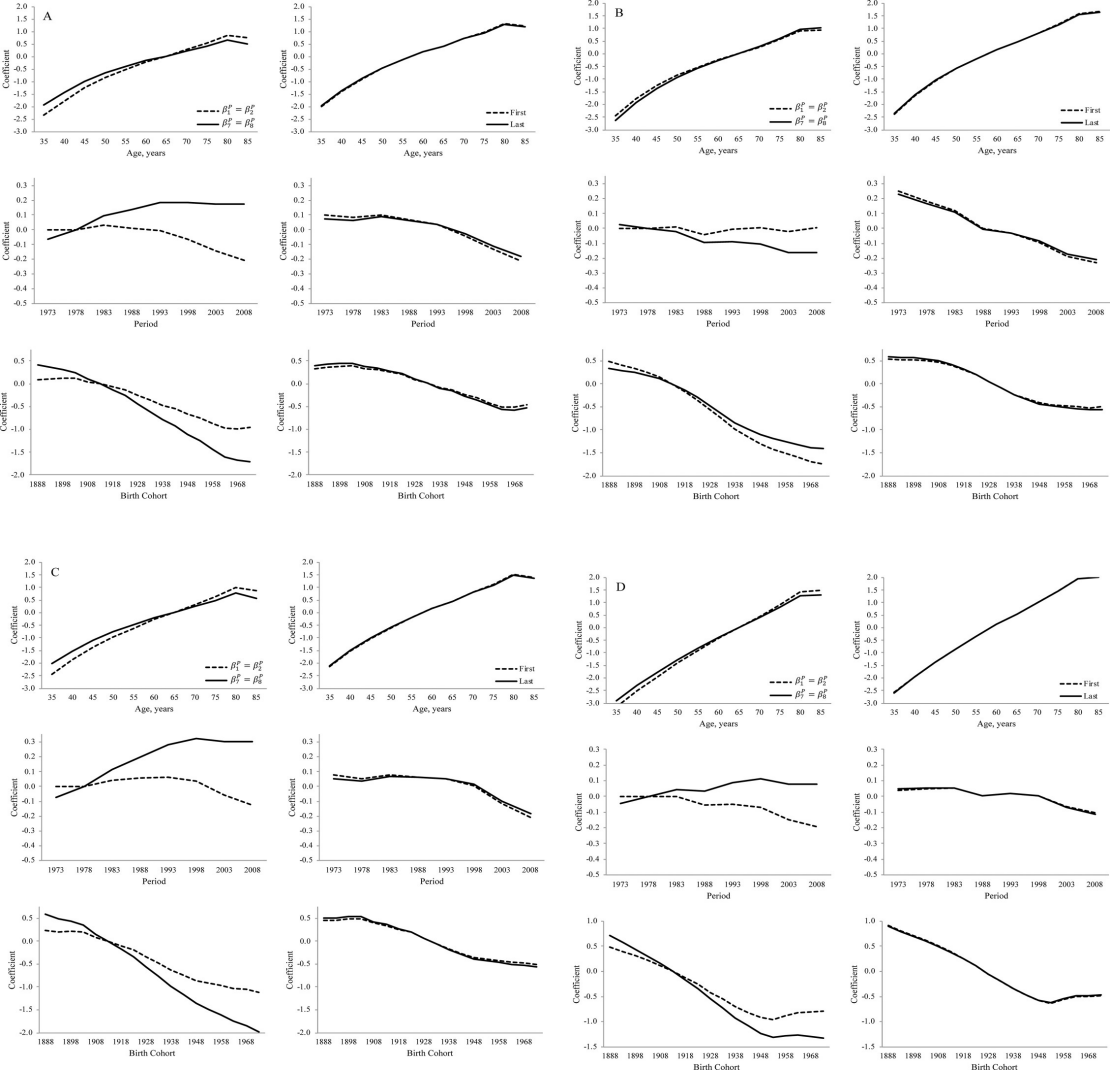
**a.** Age, period, and cohort variation in U.S. black men’s heart disease mortality rates, ages 35+ between years 1973–2010. Variations in left column estimated from CGLIM, variations in right column estimated from IE. **b.** Age, period, and cohort variation in U.S. white men’s heart disease mortality rates, ages 35+ between years 1973–2010. Variations in left column estimated from CGLIM, variations in right column estimated from IE. **c.** Age, period, and cohort variation in U.S. black women’s heart disease mortality rates, ages 35+ between years 1973–2010. Variations in left column estimated from CGLIM, variations in right column estimated from IE. **d.** Age, period, and cohort variation in U.S. white women’s heart disease mortality rates, ages 35+ between years 1973–2010. Variations in left column estimated from CGLIM, variations in right column estimated from IE.

Further, the assumption about the equality constraint receives little support in Kramer et al.’s [1] own Figs 1 and 2, which show, respectively, differences between black and white men’s and women’s period-based trends in age-standardized mortality rates and rate ratios. The heart disease mortality trends during the 1970s –the time period constrained to be equal in Kramer et al.’s [1] model (1973–1977 = 1978–1982)–appear to have differed considerably for U.S. black and white men, with faster declines observed in the white male population than in the black male population. As further evidence against the assumption justifying this constraint, see trends in age-specific heart disease mortality rate ratios between black and white women and between black and white men in S1 Fig in S1 File. Across the 1970s, the rate ratios among men increased significantly and decreased for women in five of the 11 age groups. The CGLIM APC model will estimate valid APC effects of rate ratios between black and white men and women only if the period-based variation in the ratio between black and white men was truly flat across the 1970s. The evidence suggests that this was not the case. Further, because the rate ratio between black and white women is estimated in relation to the men’s rate ratio via interaction terms, the estimated APC effects on the ratio between black and white women’s rates are likely biased as well.

To clarify and simplify constraint assumptions for readers to understand, we recommend the following:

1.a Researchers fit APC models on rates themselves, not rate ratios.
1.b Researchers fit APC models separately by population (e.g., black men, white men, black women, white women), not on pooled data using interaction terms.
1.c Researchers use APC methods that minimize subjective choice in the application of the model constraint.

Regarding point #1.c, Kramer et al.’s [1] use of the “Mason et al. method” [4] requires a researcher to set equal the variation between two ages, two periods, or two cohorts in order to identify the APC model (e.g., $\beta_{1}^{P}=\beta_{2}^{P}$). This method uses an “explicit constraint” that requires strong theory and/or empirical evidence to inform the researcher about which two parameters’ values should be constrained equal to each other in order to identify the APC model (e.g., $\beta_{1}^{P}=\beta_{2}^{P}$? or $\beta_{7}^{P}=\beta_{8}^{P}$? or $\beta_{17}^{C}=\beta_{18}^{C}$?). Several alternative APC identification strategies minimize subjective choice when applying constraints by using mechanical constraints instead. For example, the intrinsic estimator (IE) applies a Moore-Penrose (MP) generalized inverse to the singular design matrix in tabular Age x Period data. This approach builds off early work [31] that parameterizes the APC model using centered effects coding and yields a solution with smaller variance than other constrained approaches [14, 28, 30, 33–36]. Fu [30] shows the consistency of MP estimators as the number of cells in the Age x Period table increase. Other work has discussed desirable shared properties of MP estimators as well as how APC estimates can diverge depending on design matrices [37]. Additional APC methods use mechanical constraints, such as employing a maximum entropy estimator (MEE) to a bounded range of the response variable to estimate APC parameters that are set-identified [38]. This shrinkage estimator uses a probability distribution that is derived from the observed data (i.e., the range of mortality rates in the Age x Period cells) using a cross-entropy criteria. Like MP estimators, the APC estimation is information-based, but rather than providing a solution to the APC problem the technique formalizes the uncertainty in the estimates by using a measure of information entropy. Additionally, Hierarchical-Age-Period-Cohort Cross-Classified Random Effects Models (HAPC-CCREM) treat age groups as fixed effects and nest individuals in groups of time periods and birth cohorts to estimate random coefficients pertaining to period and cohort effects [14, 39–41]. Although researchers can influence model estimates by changing the number and size of the APC groupings in certain data structures (e.g., construct three-year groupings vs. five-year groupings in survey-based data), the mechanical constraints used in hierarchical APC methods and those in shrinkage estimators remain largely outside the researcher’s control [12, 14]. Constraints are not made by researchers arbitrarily setting APC model parameter values equal to one another. Instead, for example, the constraints in MP depend on the structure of the data and the minimum-norm constraint applied in the solution space [14, 30, 34, 36].

Finally, although it is known that all APC models yield biased estimates of APC parameters unless the model’s constraint exists in the population, it has been shown that some methods are better than others at minimizing this bias [13, 14, 30, 31, 42]. Overall, researchers using APC methods should make clear the constraint they use, apply it in the simplest way possible, and favor the use of methods that minimize subjective choice in the application of the constraint.

#### Test within-method consistency of APC estimates

After fitting an APC model, researchers should evaluate the sensitivity of the APC model’s estimates by changing the model’s omitted categories, constraining different model parameters, or otherwise altering model specifics [12, 14, 21, 31]. Researchers should favor results estimated from APC models that are largely invariable to the choice of constraint. Further, as Glenn [21: 20] notes, an APC method “may prove to be useful… if it yields approximately correct estimates ‘more often than not,’ if researchers carefully assess the credibility of the estimates by using theory and side information, and if they keep their conclusions about the effects tentative.” In addition to evaluating the sensitivity of APC estimates by altering the model specifics, APC researchers should also assess the consistency of the results across different populations. For example, theory and existing evidence would have us expect the estimated APC effects on black-white differences in men’s heart disease mortality to be similar to the estimated APC effects on black-white differences in women’s heart disease mortality [43–49]. The socioeconomic, institutional, and behavioral factors that are largely responsible for trends in relative black-white differences in U.S. heart disease mortality have not differed substantially for men and women across time periods or birth cohorts. Although the size of the black-white difference in heart disease mortality may differ by gender, we should not expect to see large gender-based variation in the period- and cohort-based trends in these differences. As further evidence for this expectation, trends in the age-standardized heart disease mortality rate ratios between black and white men are very similar to trends in the respective rate ratios between black and white women (see S2 Fig in S1 File). We would not expect different age-, period-, and/or cohort-based sources of variation to produce the same observed trends in women’s rate ratios as in men’s rate ratios.

Taken together, the age-, period-, and cohort-based variation in relative black-white differences in U.S. men’s heart disease mortality estimated from one specification of an APC model should be consistent with those estimated from an alternative specification of that model (i.e., demonstrate within-method consistency). And the APC effects estimated for relative black-white differences in U.S. men’s heart disease mortality should be largely consistent with the APC effects estimated for relative black-white differences in U.S. women’s heart disease mortality.

#### Test between-method consistency of APC estimates

Harper [2:1] worries about the “flexibility of APC Models” as well as the ease with which researchers can use computational routines to fit the models. Together, these concerns imply that researchers might be able to manipulate APC models to produce results that are “most consistent with their favorite hypotheses” [2: 2]. This claim can be directly tested by examining the extent to which estimated age, period, and cohort effects vary across different APC approaches. Thus, in addition to testing the within-method consistency of APC estimates by varying the omitted categories (e.g., in the case of the IE method), changing the size of the APC groupings (e.g., in the case of the HAPC-CCREM method [12]), or changing the parameter equality constraints (e.g., in the case of CGLIMs), researchers should validate their APC estimates by comparing them to estimates from alternative APC methods. That is, directly estimate the variation in APC model estimates across different methods. Researchers should favor results from APC models that are most consistent across multiple methods.

## Materials and methods

### Data

To demonstrate the utility of the guidelines above, we revisit Kramer et al.’s [1] analyses of heart disease mortality trends among U.S. black and white men and women. Deaths caused by heart disease were coded from ICD-8 for deaths in 1973–1978 (390–398, 402, 404, and 410–429) from ICD-9 for deaths in 1979–1998 (390–398, 402, and 404–429) and from ICD-10 for deaths in 1999–2010 (I00-I09, I11, I13, and I20-I51). Analytic data from Kramer et al.’s Table 1 [1] are presented again as Table 1 below. These data are official U.S. mortality rates for years 1973–2010 made available from CDC Wonder [50]. Five-year age-specific mortality rates from heart diseases (11 age groups 35–39, …, 85+) across seven five-year time periods (1973–1977, …, 2003–2007) and one three-year time period (2008–2010) are shown for U.S. non-Hispanic black and white men and women. From the data in Table 1, 18 10-year birth cohorts can be calculated as linear outcomes of Period-Age = Cohort (-1892, 1888–1897, …, 1963–1972, 1968–1975).

**Table 1:** U.S. heart disease mortality rates by race, gender, age, and period, 1973–2010.

	1973–1977	1978–1982	1983–1987	1988–1992	1993–1997	1998–2002	2003–2007	2008–2010
**Black Women**								
35–39	49.9	41.5	36.1	33.5	34.3	30.0	27.6	24.5
40–44	97.4	82.7	73.3	66.9	63.6	59.1	51.3	46.6
45–49	178.5	153.3	132.2	123.2	113.9	100.9	88.7	78.9
50–54	295.4	260.1	239.8	215.0	193.6	160.0	144.5	126.9
55–59	455.3	419.8	398.8	357.0	320.1	267.0	213.7	186.7
60–64	725.1	660.9	645.1	584.4	504.5	422.7	332.3	279.3
65–69	1043.1	954.1	934.8	879.9	761.6	647.2	493.6	401.7
70–74	1751.7	1484.3	1460.9	1289.3	1221.6	1001.8	762.1	618.3
75–79	2272.8	2177.2	2098.7	1941.1	1743.4	1574.9	1219.7	999.6
80–84	3237.2	3418.4	3447.3	3062.2	2811.5	2444.9	2033.7	1662.6
85+	2920.8	2854.9	2921.5	2946.6	2671.1	2462.9	2091.5	1734.0
**White Women**								
35–39	13.3	11.8	10.0	8.4	9.6	10.2	9.8	9.5
40–44	28.8	25.5	21.5	16.5	17.1	17.7	18.7	17.6
45–49	55.4	50.5	43.9	34.9	32.9	30.1	32.0	31.5
50–54	105.4	95.4	87.8	70.7	64.4	55.6	50.8	50.2
55–59	198.6	179.0	163.8	139.2	122.3	104.0	85.1	76.1
60–64	370.0	336.2	308.3	255.0	230.5	189.3	151.7	129.3
65–69	648.8	592.5	540.6	443.0	390.4	331.7	252.8	211.1
70–74	1168.7	1054.0	962.6	791.9	690.7	577.8	453.6	368.2
75–79	2112.8	1866.3	1693.0	1413.2	1236.3	1049.1	831.5	691.1
80–84	3668.0	3395.8	3093.0	2637.7	2343.5	2025.0	1632.0	1343.4
85+	4381.1	3974.1	3568.3	3120.7	2864.0	2532.3	2087.0	1753.8
**Black Men**								
35–39	104.4	93.3	85.0	74.8	63.3	54.2	53.4	50.3
40–44	197.5	186.5	172.4	142.8	126.8	104.9	92.4	83.4
45–49	370.4	335.9	292.9	271.7	241.5	194.3	172.0	145.1
50–54	596.6	549.0	510.2	439.4	406.8	325.7	295.4	245.4
55–59	854.0	844.5	757.4	698.3	618.1	529.9	441.1	385.5
60–64	1253.9	1185.6	1184.6	1034.9	929.8	769.7	647.3	550.7
65–69	1663.3	1585.0	1570.3	1459.1	1247.6	1066.4	870.6	774.8
70–74	2482.7	2229.8	2277.9	2033.3	1905.8	1570.2	1254.7	1046.7
75–79	3138.6	2993.9	2949.2	2786.4	2502.5	2233.7	1847.4	1566.1
80–84	4310.6	4483.4	4548.5	4065.4	3770.1	3274.0	2784.9	2340.7
85+	3748.9	3813.8	3785.7	3805.0	3529.0	3277.0	2906.4	2506.0
**White Men**								
35–39	47.9	41.4	35.7	27.5	27.3	24.9	24.1	22.5
40–44	117.8	95.9	81.1	62.6	57.6	53.3	50.7	45.2
45–49	240.3	201.3	162.3	128.5	113.9	99.3	93.0	86.0
50–54	419.9	358.2	303.5	230.6	205.6	169.4	155.0	144.4
55–59	695.4	595.9	509.2	404.0	340.3	278.7	234.6	217.3
60–64	1107.6	944.0	818.9	654.2	567.2	445.6	360.6	315.7
65–69	1663.7	1462.6	1262.7	1014.1	869.7	695.6	530.2	453.1
70–74	2452.0	2220.7	1992.2	1582.1	1350.5	1102.4	835.5	695.5
75–79	3673.4	3321.2	3018.8	2461.2	2132.1	1783.3	1385.9	1153.9
80–84	5455.2	5080.5	4700.9	3993.3	3549.9	3030.7	2443.9	2043.0
85+	6014.1	5510.8	5029.3	4490.8	4249.9	3801.1	3203.6	2744.2

We also obtained counts of death from heart disease for single-year ages (35–74) from the National Center for Health Statistics (NCHS) for each year 1970–2009 and mid-year population counts for single-year age (35–74) from the National Cancer Institute’s Surveillance, Epidemiology, and End Results (SEER) Program’s U.S. Population Data [51]. These NCHS-SEER data were then collapsed into 12 three-year age groups (39–41, …, 72–74) and one four-year age group (35–38) in order to generate descriptive heat plots in relative black-white mortality rate ratios across single year periods 1970 through 2009.

The mortality rates in Table 1 can be analyzed with a general APC model specified as:

$$\log\;E(r_{ij})=\log\;E\left(\frac{d_{ij}}{n_{ij}}\right)=\beta_{0}+\beta_{i}^{A}+\beta_{j}^{P}+\beta_{k}^{C},$$ (1)

where log *E*(*r*_*ij*_) is the logarithm of the expected heart disease mortality rate based on *d*_*ij*_ deaths and *n*_*ij*_ person-years in cell *ij* of a cross-classification of deaths and person-years in age interval *i* (for *i* = 1,…, *I* age groups) and time period *j* (for *j* = 1,…, *J* periods). Age and period effects are denoted by $\beta_{i}^{A}$ and $\beta_{j}^{P}$, respectively. $\beta_{k}^{C}$ denotes the *k*th (diagonal) birth cohort effect (for *k* = 1, …, *I+J*-1 birth cohorts), where the index *k* = *I–i* + *j*. For these data, *I* = 11 and *J* = 8 for *N* = *I* x *J* = 88 age x period cells covering 18 birth cohorts. (see S1 Table in S1 File for an illustration of the Age x Period data structure)

In the exercise below, we follow the above guidelines using a) the CGLIM *coefficients-constraint approach* employed by Kramer et al. [1] as well as b) adopting the *IE approach* as an alternative APC method to serve as a comparison case. Consistent with guideline point #1, compared to the CGLIM, the IE has a number of attractive properties for examining age-, period-, and cohort-based variation in the U.S. heart disease mortality rates observed in Table 1. The data are tabulated age-specific rates across time periods and satisfy the perfect linear dependency between age, period, and cohort [14]. Further, the IE constraint is a function of the design matrix (i.e., the data structure of 11 Age groups x 8 Period groups = 88 cells) and is not determined by our manipulation of APC model parameters via equality constraints. Thus, the mechanical constraint imposed by the IE is more in line with guideline point #1.c than is the CGLIM, as the latter’s explicit constraint manipulates the value of model parameters and the former’s constraint is a function of the design matrix. To follow points #1.a and #1.b in the guidelines, we first fit the APC models separately to mortality rates among U.S. black and white men and women.

We then follow point #2 in the guidelines by examining the extent to which APC coefficients estimated from a CGLIM identified via the *coefficients-constraint approach* differ when the model is refitted using an alternative parameter constraint. Specifically, we fit one CGLIM using the equality constraint employed by Kramer et al. [1], $\beta_{1}^{P}=\beta_{2}^{P}$, and fit another CGLIM using an equality constraint on the last time periods, $\beta_{7}^{P}=\beta_{8}^{P}$. We also examine the extent to which the APC coefficients estimated from the IE model omitting the first APC category levels in the effect coding differ from the APC coefficients estimated from IE model omitting the last APC category levels. That is, we test within-method consistency in the CGLIM approach and within-method consistency in the IE approach.

We follow point #3 in the guidelines in two ways. First, we compare APC coefficients for U.S. white men’s heart disease mortality estimated from CGLIMs and IE models with coefficients estimated from APC models using set identification with MEE, HAPC-CCREMs, and Bayesian APC models (BAM) [52, 53]. Second, we estimate APC variation in relative black-white differences in heart disease mortality among U.S. women and men using the CGLIM, the IE, the MEE, and the HAPC-CCREM. That is, we examine between-method discrepancies in the estimates of APC variation in relative black-white differences in heart disease mortality, as this was the central aim of Kramer et al.’s investigation [1].

We imagine a scenario in which eight separate research teams were tasked with using APC analyses to examine cohort trends in the relative black white differences in U.S. heart disease mortality. Let us say that four research teams did not follow the guidelines and used the CGLIM approach with equality constraints. Because the teams did not follow the guidelines, it is possible that the four teams adopted four different equality constraints: $\beta_{1}^{P}=\beta_{2}^{P},\;\beta_{4}^{P}=\beta_{5}^{P},\;\beta_{1}^{C}=\beta_{2}^{C}$, and $\beta_{17}^{C}=\beta_{18}^{C}$. That is, the teams may have drawn from different sources of side information, theory, and/or different interpretations of an “APC toolbox” to arrive at various equality constraints [3]. For example, constraints on the last two birth cohorts, $\beta_{17}^{C}=\beta_{18}^{C}$, might have been motivated by existing evidence and theory suggesting that declines in U.S. heart disease mortality may be slowing or stalling as a result of the U.S. obesity epidemic [46, 54–56]. And constraints on the middle two time periods might have been justified by the research team observing the black-white rate ratios for women and men beginning to flatten across these times. (See again the trends in age-standardized black-white heart disease mortality rate ratios in S2 Fig in S1 File).

In contrast, we imagine the other four research teams adopted and followed the guidelines. As a result, each research team opted to fit APC models that employ mechanical constraints to minimize their influence on the estimates. Let us say two teams used the IE approach, with one omitting the first APC category levels $\left(\beta_{1}^{A},\beta_{1}^{P},\beta_{1}^{C}\right)$ and the other omitting the last APC category levels $\left(\beta_{11}^{A},\beta_{8}^{P},\beta_{18}^{C}\right)$ for identification, one team used set identification with MEE, and one team used HAPC-CCREM.

We contrast the APC patterns in the rate ratios estimated from the set of four CGLIMs with patterns in the rate ratios estimated from the two IEs, the MEE, and the HAPC-CCREM. Taken together, the exercise provides evidence to a) evaluate Kramer and Casper’s [3] claim that a general “APC toolbox” exists for cohort analyses, b) test Harper’s [2: 2] claim that researchers can manipulate APC models to produce results that are “most consistent with their favorite hypotheses,” and c) evaluate the effectiveness of the guidelines at resolving differences in APC results. If a general “APC toolbox” exists, then we should observe APC results estimated from the four CGLIMs and estimated from the four other APC models to be generally consistent with each other. Conversely, if Harper’s claim is accurate, then we should observe APC results estimated from all eight models to be at odds with each other. That is, APC estimates should vary widely across the approaches, giving us an array of results from which to choose. Finally, the guidelines would lead us to expect discordance among the APC patterns estimated from the CGLIMs and consistency in the APC patterns estimated from the IE, the MEE, and the HAPC-CCREM. The constraints used in each of these approaches are briefly discussed in the appendix, which also includes analytic scripts in R [57], WinBUGS [58], and Stata [59].

## Results

Black-white mortality rate ratios from heart disease deaths are arrayed in percentiles across age groups (35–39, 40–42, …, 72–74) and time periods (1970, …, 2009) in Fig 1.

Darker shades indicate relatively larger rate ratios and lighter shades indicate relatively smaller ratios. Age, period, and cohort patterns in the rank percentiles can be gleaned by observing the extent to which the shades become lighter across the y-axis (i.e., age), across the x-axis (i.e., period) and across diagonals (i.e., birth cohort). Among both U.S. men and women, we observe relative black-white differences in heart disease mortality to narrow with age (i.e., the darkest shades tend to be concentrated at the youngest ages and the lightest shades are observed at the oldest ages). If we start in the top-left corner of the figures (i.e., the earliest birth cohorts) and follow downward across the diagonals toward the bottom-right corner of the figures (i.e., the most recent birth cohorts), we observe that the shades tend to darken across subsequent cohorts. The shades also lighten in the very most recent cohorts. These patterns indicate that relative black-white differences in U.S. heart disease mortality likely widened across most birth cohorts in the twentieth century, but may have narrowed among more recent birth cohorts. Finally, period-based trends in black-white differences are more difficult to observe in men’s heart disease mortality. No clear pattern in the shading is evident across the x-axis, especially at the younger ages (e.g., 35–59). The shading among older ages (e.g., 60+) tends to darken across time periods, but these trends are difficult to attribute separately to period- or cohort-based sources. Period-trends also are difficult to observe among women, but relative black-white differences in heart disease mortality do appear to have narrowed across recent time periods. Specifically, the shades appear to lighten for all age groups across the late-1990s and 2000s. Overall, the APC patterns in relative black-white differences in U.S. heart disease mortality descriptively appear to be quite similar for men and women. Black-white differences narrow across age, widened across birth cohorts, and were relatively stable across time periods albeit with some recent narrowing appearing to have occurred for women. The empirical “side information” provided by these descriptive plots can be helpful in assessing estimates from statistical APC models [21].

Point estimates of APC coefficients estimated from the CGLIM using the equality constraint $\beta_{1}^{P}=\beta_{2}^{P}$ and estimated from the CGLIM using equality constraint $\beta_{7}^{P}=\beta_{8}^{P}$ are shown in the left-hand panels of Fig 2A–2D. Also included in Fig 2A–2D (right-hand panels) are APC coefficients estimated from two models fitted using the IE. One IE model was fitted by omitting the first APC category levels in the effect coding (i.e., age 35–39, period 1973–1977, cohort 1888–1892) and the other model was fitted by omitting the last APC category levels (i.e., age 85+, period 2008–2010, cohort 1973–1977).

APC coefficients estimated from the CGLIM fitted by using Kramer et al.’s [1] constraint, $\beta_{1}^{P}=\beta_{2}^{P}$, are substantively different from the APC coefficients estimated by the CGLIM that constrained the last two period parameters, $\beta_{7}^{P}=\beta_{8}^{P}$. The discrepant APC estimates are seen in all populations and are especially large for estimates of period and cohort effects on heart disease mortality. Consistent with others’ warnings, APC coefficients estimated from CGLIMs appear to be highly sensitive to a researcher’s choice of the coefficient constraint [14, 21, 35]. In contrast, the sets of APC coefficients estimated from the two IE models are very much consistent with each other in all populations. That is, the age-, period-, and cohort-based variation in black and white men’s and women’s heart disease mortality rates estimated from the IE model are robust to changes in model specification. Contrary to Harper’s [2] concern that researchers can specify APC models to produce results supportive of their favorite hypotheses, APC estimates from the IE models are invariant to the choice of the omitted APC categories in the models’ centered effects coding.

In their online supplement, Kramer et al. [1] test between-method consistency of APC estimates by plotting mortality rate ratios estimated from three different APC methods: the CGLIM method using the equality constraint, $\beta_{1}^{P}=\beta_{2}^{P}$, the IE, and the Median Polish approach [24, 60]. Kramer et al.’s comparison makes assessing the consistency of the models’ results difficult because they compare *between-population estimates within a method* (e.g., they plot the IE method’s estimated APC rate ratios separately for white men, for black men, for white women, and for black women together in one graph). A more useful comparison of between-method results would be to contrast within-population estimates from different methods (e.g., plot the APC effects on black women’s heart disease mortality rates estimated from the IE method, the median polish method, and the CGLIM method on the same graph). Because we are concerned with between-method variation in the APC estimates, researchers should make the comparison of alternative methods’ estimates as easy to gauge as possible.

**Fig 3:**
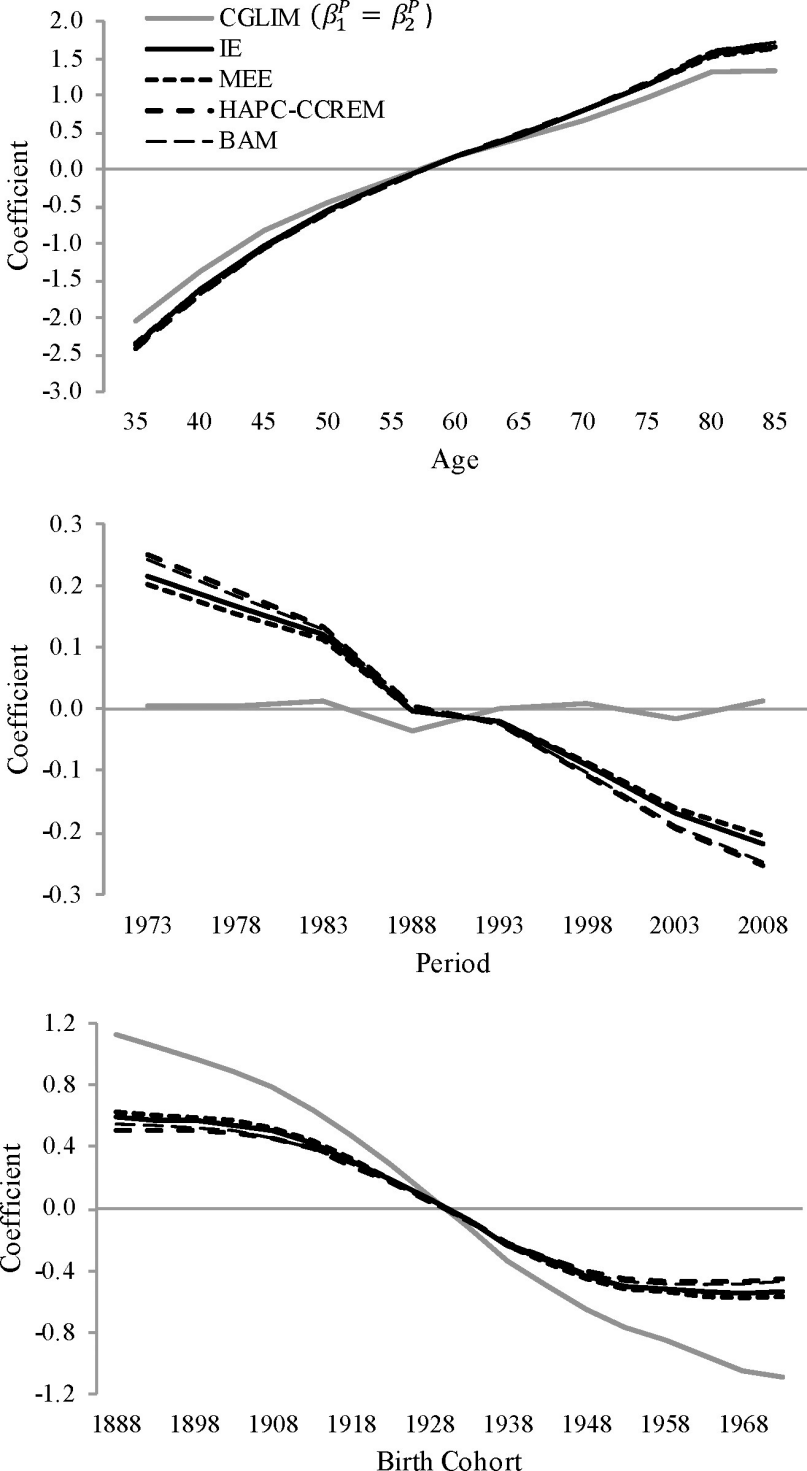
Normalized point estimates of APC coefficients for U.S. white men’s heart disease mortality rates, ages 35+ between years 1973–2010, estimated from APC models using CGLIM with a $\beta_{1}^{P}=\beta_{2}^{P}$ constraint, IE constraint, MEE constraint, HAPC-CCREM-MCMC constraint, and BAM constraint.

In this spirit, Fig 3 plots point estimates of APC coefficients for white men’s heart disease mortality rates estimated from APC models using five different constraints: a) Kramer et al.’s CGLIM that constrains the first two periods, $\beta_{1}^{P}=\beta_{2}^{P}$, b) the average of two models using the IE constraint, one omitting the first categories of APC and the second omitting the last categories of APC, c) the MEE, d) HAPC-CCREMs estimated from MCMC simulations, and e) Bayesian APC models (BAM) using the bamp package in R [53]. Results show that the age, period, and cohort coefficients estimated from models using the IE, MEE, HAPC-CCREM, and BAM constraints are consistent with one another. From all models, we see estimated age-based increases in U.S. white men’s heart disease mortality and large declines across both time periods and birth cohorts, albeit with slowing declines and flat trends across recent cohorts. The latter findings are consistent with existing evidence suggesting cohort-based stalling in U.S. heart disease mortality and large period-based declines across this time [42, 54, 56].

In Fig 3, we also see that the CGLIM using the equality constraint yields APC coefficients that are inconsistent with these patterns. The age patterns are more attenuated than the age-based patterns suggested by the four other models, no period-based variation is estimated, and cohort-based declines are estimated to be much larger than the declines estimated from the other models. Overall, the results show that four different APC modeling strategies (i.e., the IE, MEE, HAPC-CCREM, and BAM) estimate very similar APC variation in U.S. white men’s heart disease mortality, while the CGLIM estimates APC patterns that seriously conflict with the other models.

Points #2 and #3 in the guidelines advise that we should favor the APC patterns estimated from the IE over the results estimated from the CGLIM. Regarding point #2, the estimates from the IE model are internally consistent and robust to model specification. Specifically, omitting the first APC categories versus omitting the last APC categories in the models’ effect coding did not significantly or substantively alter the estimates of the APC coefficients. Conversely, APC patterns estimated from the CGLIMs varied considerably depending on our choice of equality constraint. Regarding point #3, the APC patterns in U.S. white men’s heart disease mortality rates estimated from the IE are consistent with the patterns estimated from two other APC approaches. In contrast, the APC patterns estimated from the CGLIM using the equality constraint $\beta_{1}^{P}=\beta_{2}^{P}$ seriously conflict with the other models’ results.

The consistent APC patterns estimated by the IE, MEE, HAPC-CCREM, and BAM counter Harper’s [2: 1] worry that different APC methods can produce “wildly different conclusions.” The IE estimates also provide evidence against his notion that researchers can strong-arm APC models into generating results that are “most consistent with their favorite hypotheses” (2). The evidence also supports our earlier suggestion that researchers favor APC methods that minimize the use of arbitrary and subjective constraints. Specifically, we see that the four APC methods that use mechanical constraints–the IE, the MEE, HAPC-CCREM, and BAM–estimated APC coefficients that are quite consistent with one another. Conversely, Kramer et al.’s [1] identification strategy of forcing equal variation in heart disease mortality during the 1973–1978 and 1978–1983 time periods estimated very different results that are sensitive to the equality constraint.

**Fig 4:**
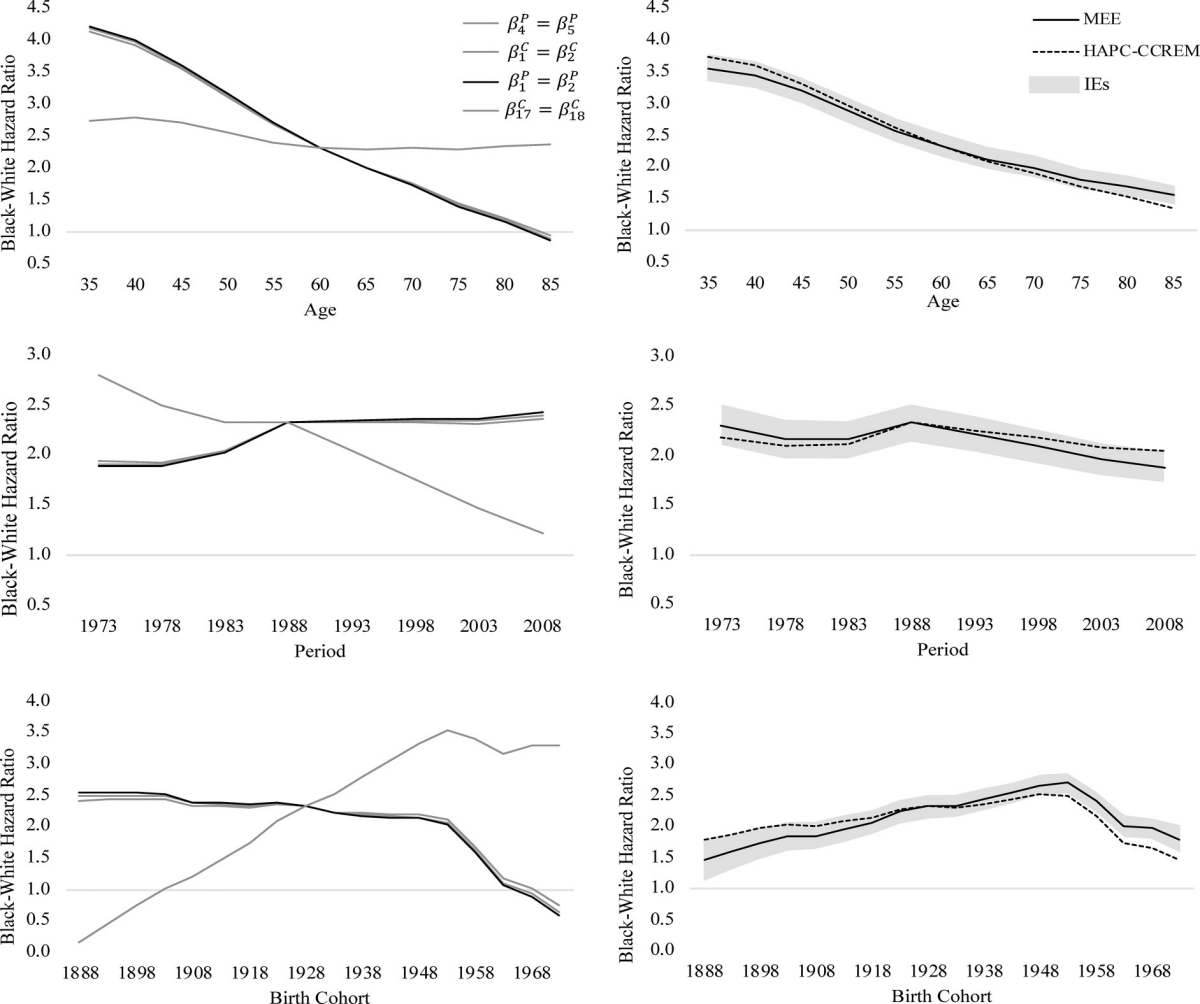
**Age, period, and cohort variation in relative black-white differences in U.S. women’s heart disease mortality, ages 35+ between years 1973–2010, estimated from APC models using CGLIM with equality constraints (left) and estimated from APC models using the IE, MEE, and HAPC-CCREM-MCMC (right).** Note: Shaded area denotes 95% CI for the IE estimates.

**Fig 5:**
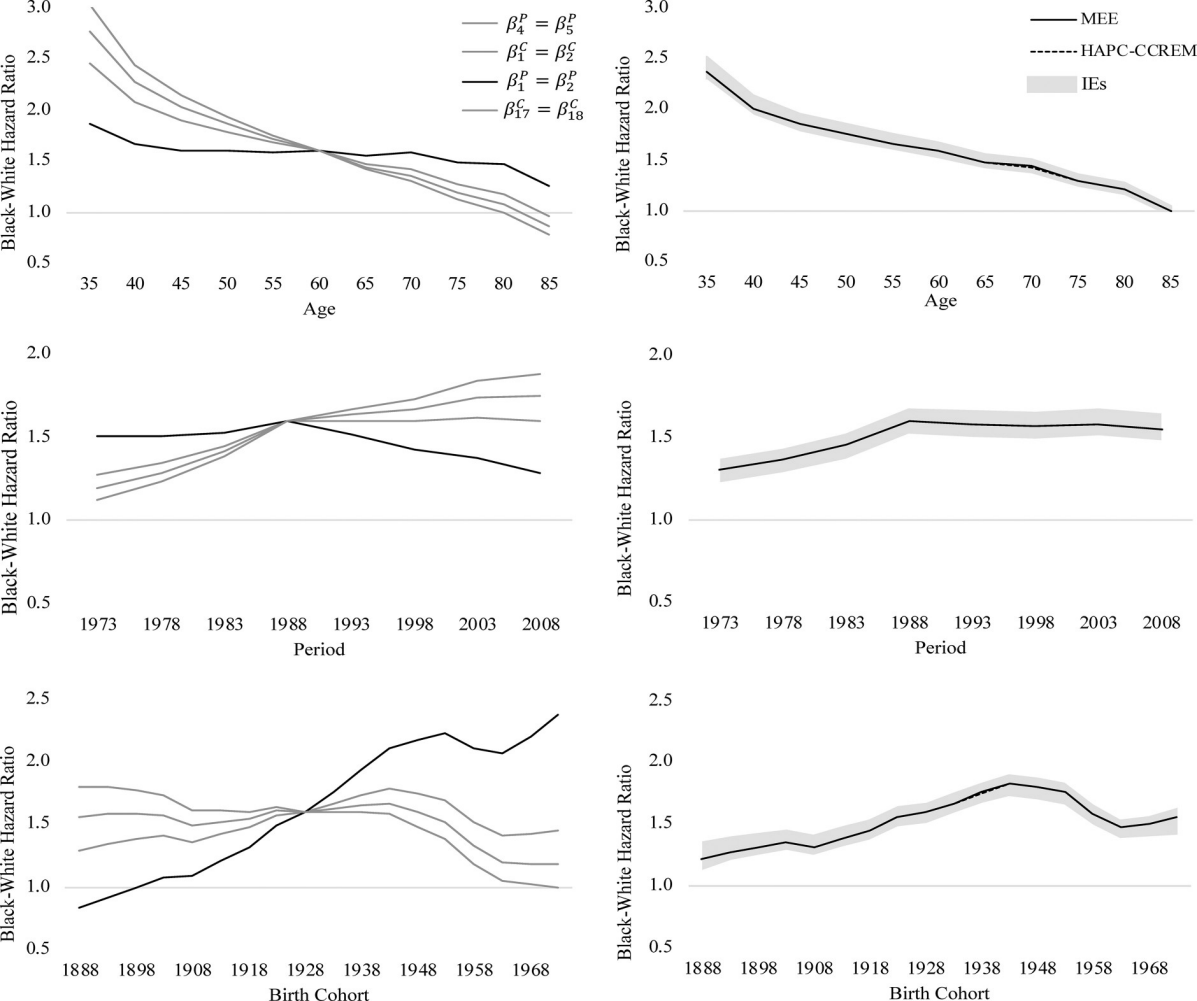
**Age, period, and cohort variation in relative black-white differences in U.S. men’s heart disease mortality, ages 35+ between years 1973–2010, estimated from APC models using CGLIM with equality constraints (left) and estimated from APC models using the IE, MEE, and HAPC-CCREM-MCMC (right**). Note: Shaded area denotes 95% CI for the IE estimates.

Finally, we review the eight hypothetical research teams’ results about cohort trends in relative black-white differences in U.S. heart disease mortality. In the left-hand panels of Figs 4 (women) and 5 (men), we plot the black-white rate ratios estimated from the four CGLIMs. In the right-hand panels of Figs 4 and 5, we plot the respective black-white rate ratios estimated from models identified using the IE, the MEE, and the HAPC-CCREM. The rate ratios across time periods are estimated at age 60–64 and birth cohort 1928–1932, the rate ratios across birth cohorts are estimated at age 60–64 and time period 1988–1992, and the rate ratios across ages are estimated at time period 1988–1992 and birth cohort 1928–1932. These values were chosen because they are closest to the average age, period, and cohorts in these data.

We assess the results using the two criteria from the guidelines (i.e., within-method consistency and between-method consistency) as well as existing “side information” [21] via both theory and evidence on U.S. black and white heart disease mortality trends. The APC patterns estimated by Kramer et al.’s CGLIM are shown to differ greatly from the ratios estimated by the CGLIM constraining the other APC parameters. The extent of the variability also differs by men and women. In Fig 4, we see that three of the CGLIMs estimate consistent APC patterns in black-white mortality ratios for U.S. women, but one of the CGLIMs estimates patterns that are strikingly different from the others (i.e., very little age-based variation and very large cohort-based increases in black-white differences). In Fig 5, we see that all four of the CGLIMs estimate widely varying APC patterns in the black-white differences among U.S. men. As such, these four research teams could indeed arrive at “wildly differing conclusions” about cohort-based trends in heart disease mortality [2: 2]. Moreover, the within-method consistency among three of the CGLIMs’ APC estimates among U.S. women could falsely assure researchers that their model estimates are valid. This is a good example highlighting the need to further validate APC model results by assessing between-method consistency in model estimates.

Between-method consistency in the APC results estimated from the CGLIMs can be assessed by reviewing the estimated APC patterns in the right-hand panels of Figs 4 and 5. The APC patterns in relative black-white differences in U.S. heart disease mortality estimated from the two IEs, the MEE, and the HAPC-CCREM are quite consistent with each other, but diverge strongly from the patterns estimated by the CGLIMs. Among the APC patterns in the right-hand panels, we see black-white differences are estimated to narrow with age in ways that are similar for men and women. Further, little period-based variation is estimated, although differences among U.S. women did narrow some across recent periods. Finally, relative black-white differences in heart disease mortality are estimated to have widened across birth cohorts born during the early twentieth century and then narrowed across more recent cohorts. The estimated cohort-based trends are similar for men and women and consistent with existing evidence on cohort-based trends in U.S. heart disease mortality [46, 54, 61].

Finally, we can assess the APC patterns estimated from the various models by comparing them to descriptive results in Table 1 and Fig 1. The “side information” provided from these descriptive sources can help identify questionable results estimated from APC models. From Table 1, for instance, we see that the relative black-white differences in heart disease mortality rates range from 2.5 to 4.0 among younger women and ranges from 1.7 to 2.7 among younger men. Also, the ratios range from 0.7 to 1.3 among older women and range from 0.6 to 1.2 among older men. Further, the patterns in Fig 2 indicated strong age-based variation in the rate ratios for both men and women. From this information, we should expect the age patterns in the black-white rate ratios estimated from the APC models to be consistent with these ranges and to decline with age. Many of the age patterns estimated by the CGLIMs do not meet these expectations. Among women, three of the CGLIMs estimate ratios at the youngest ages that exceed the observed rate ratios in Table 1 (e.g., 4.2), and one CGLIM estimates no substantive age-based variation in the rate ratios. Ratios at the older ages estimated from this latter model are inconsistent with the observed ratios in Table 1 (e.g., 2.4). Among men, two of the CGLIMs estimate ratios at the youngest ages that exceed the observed ratios in Table 1 (e.g., 2.8–3.0) and the model reported by Kramer et al. [1] estimates very little age-based variation in the rate ratios. Ratios at the older ages estimated from this CGLIM are inconsistent with the range of ratios observed in Table 1 (e.g., 1.3–1.5 estimated vs. the .6–1.2 observed). Conversely, the age patterns in the black-white rate ratios estimated from the IEs, the MEE, and the HAPC-CCREM are all consistent with the observed ratios in Table 1 as well as the descriptive patterns observed in Fig 1. Moreover, the period- and cohort-based variation estimated from these models are also consistent with the trends observed in Fig 1 and are similar for men and women.

To recap, 1) the APC patterns in the rate ratios estimated from the IEs are robust to alternative model specification whereas the respective patterns estimated from the CGLIMs vary considerably depending on the equality constraint, 2) the APC patterns estimated from the IEs match those estimated from the MEE and the HAPC-CCREM whereas the CGLIMs estimated erratic APC patterns that are inconsistent with the other models’ estimates, 3) the APC patterns estimated from the IEs are consistent with observed APC patterns in descriptive tables and graphs whereas the CGLIMs estimated APC patterns that exceeded rate ratio values in observed data and/or seriously conflicted with the patterns observed in descriptive plots, and 4) the APC patterns estimated from the IEs were similar among men and women whereas the patterns reported by Kramer et al. [1] from their CGLIMs differ remarkably for men and women. By all criteria in the guidelines, we should favor the APC patterns in relative black-white differences in heart disease mortality that were estimated from the IE models and seriously question the patterns that were estimated from the CGLIMs.

## Discussion

Researchers aiming to document period and cohort trends in social and health outcomes will likely continue to use APC models in their studies. Some researchers will strongly caution against the use of APC models, while others will perhaps defend and even advocate their use. We urge readers to consider that the best practice for cohort analyses is to cautiously assess the strengths and weaknesses of different APC approaches, as some techniques are more advantaged than others for identifying cohort-based variation in outcomes. Although Kramer and colleagues [1, 3] and Harper [2] both acknowledged that APC models are at best descriptive tools, neither recognized the important differences that exist across APC approaches for estimating cohort variation in outcomes. On the one hand, Kramer and colleagues advocated the use of a general “APC toolbox.” On the other hand, Harper [2: 2] suggested “that APC models should be used with caution,” but his indiscriminate warning did not distinguish between various APC approaches. Given the difficulties and potential pitfalls in APC analyses, researchers need to recognize that not all APC approaches are the same–they constitute neither a “toolbox” for easy application nor a black box susceptible to easy manipulation.

In this paper, we reviewed and tested easy-to-implement steps for the cautious use of APC models, which build upon others’ suggestions for APC researchers to follow (e.g., [13, 14]). To recap, we encourage APC researchers to first use descriptive tables and graphs as exploratory exercises. Plot age-specific or age standardized rates across time periods in order to identify possible “non-parallelism” in the trends [13]. If the trends appear to be parallel, it is likely that a reduced age-period (AP) model or age-cohort (AC) model will be preferred to a full APC accounting model [12–14, 42]. As advised by others, researchers can also use GOF statistics to help assess if APC models likely account for more variation in the outcome than do simpler AP or AC models [14]. An APC model fitted to data where a simpler model is preferred will estimate biased results [8, 12, 14, 42]. Descriptive plots such as “heat maps” should also be used to visually assess apparent age, period, and/or cohort patterns in outcomes. If evidence from these preliminary analyses indicate that your outcome likely varies by age *and* period *and* cohort, then your analyses may benefit from fitting a full APC statistical model. If so, then:

1. Simplify models and clearly state your assumptions.
  - Fit APC models on counts of deaths or rates, not rate ratios.
  - Fit APC models separately by subpopulations, not on pooled data using interaction terms.
  - Favor APC methods that minimize the degree of subjective choice in the selection and application of constraint.
2. Test within-method consistency of APC estimates (i.e., specify the model differently and compare the estimates to those from the original model)
3. Test between-method consistency of APC estimates (i.e., compare model results to those estimated from models identified by using different constraints)

Using these guidelines to assess the validity of Kramer and colleagues’ “APC toolbox,” we recap the following conclusions: a) the research team took a “somewhat unorthodox approach” to constrain the model, making the identification strategy difficult to follow [2: 1], b) the CGLIM approach required the researchers impose an arbitrary equality constraint on the model parameters that appears to receive little empirical or theoretical support, c) when the CGLIM was fitted with a different equality constraint, the APC estimates differed substantially from those reported by Kramer et al. [1], d) the CGLIM estimated APC patterns that were inconsistent with patterns estimated by several alternative APC methods, e) the CGLIM estimated APC patterns that were in conflict with the rates and ratios observed in descriptive data, and f) the CGLIM estimated APC patterns in the relative black-white differences in heart disease mortality among men that differed substantially from the APC patterns among women. These contradictory results and shortcomings to the CGLIM approach are strong evidence against the existence of a general “APC toolbox.” Indeed, if Kramer and colleagues has used the guidelines reviewed here to inform their analytic approach and to critically assess their results, they likely would have arrived at very different conclusions about APC trends in black-white differences in U.S. heart disease mortality.

Yet, by applying the guidelines to Kramer et al.’s data and results, we also found little support for Harper’s [2: 2] contentions that 1) APC models arrive at “wildly differing conclusions regarding the influence of period and cohort effects” or 2) that “researchers could end up choosing APC models that are most consistent with their favorite hypotheses.” On the contrary, when we adopted the guidelines and used constraints that are not affected by subjective decisions, APC estimates of black-white differences in heart disease mortality were consistent across the models and the results were the same for U.S. men and women. Moreover, estimates from the IE models were robust to the selection of the omitted APC categories in the models’ centered effects coding.

## Conclusion

By adopting standard guidelines for APC analyses, researchers can likely move conversations about APC methods beyond sweeping criticisms and/or support for generic “APC toolboxes” toward more practical discussions about how researchers using APC methods should articulate assumptions, specify multiple models, and critically assess their results. The exercises here demonstrated the simplicity and effectiveness of following such guidelines in resolving disagreements over APC results. The cautious use of APC models can generate results that are consistent across methods and robust to researcher manipulation.

## Supporting information

S1 File (DOCX)

## Abbreviations

APC: Age-Period-Cohort
AP: Age-Period
AC: Age-Cohort
U.S: United States
CGLIM: Constrained Generalized Linear Model
MP: Moore-Penrose
IE: Intrinsic Estimator
MEE: Maximum Entropy Estimator
HAPC-CCREM: Hierarchical Age-Period-Cohort Cross-Classified Random Effects Model
MCMC: Markov chain Monte Carlo
BAM: Bayesian Age-Period-Cohort Model
ICD: International Statistical Classification of Disease and Related Health Problems
CDC: Centers for Disease Control and Prevention
GOF: Goodness of Fit
