# Supplementary material for: Clarifying assumptions in age-period-cohort analyses and validating results
Source: PLoS One. 2020 Oct 6;15(10):e0238871. doi: 10.1371/journal.pone.0238871 (PMC7537862; doi:10.1371/journal.pone.0238871)
Supplement: S1 File — (DOCX) [file pone.0238871.s001.docx]

Supplementary Material

1. Supplementary Table 1: Age x Period data structure.
2. Review of age-period-cohort models’ various constraints.
3. Supplementary Figure 1: trends in age-specific black-white heart disease mortality rate ratios
4. Supplementary Figure 2: trends in age-standardized black-white heart disease mortality rate ratios
5. Stata code for APC models using IE, CGLIM, and MEE constraints fitted to U.S. white men’s heart disease mortality rates.
6. R code for HAPC-CCREMs using MCMC simulations fitted to U.S. white men’s heart disease mortality rates.
7. Samples of OpenBUGS output for HAPC-CCREMs using MCMC simulations fitted to U.S. white men’s heart disease mortality rates.
8. R code for Bayesian APC models using bamp package.

**Age x Period Data Structure**

Table 1 in the manuscript presents official U.S. mortality rates for years 1973-2010. Five-year age-specific mortality rates from heart diseases among black and white men and women are presented by five-year age groups (11 groups 35-39, …, 85+) across seven five-year time periods (1973-1977, … , 2003-2007) and one three-year time period (2008-2010). From the data in Table 1, 18 10-year birth cohorts can be calculated as linear outcomes of Period-Age = Cohort (1883-1892, 1888-1897, …, 1963-1972, 1968-1975). The structure of these data is shown on the following page using single-year age and single-year period with the age groupings indicated with blue and white and period groupings indicated with yellow and white. The 10-year cohort groupings are the diagonals.

**Supplementary Table 1**. Illustration of the Age x Period Data Structure: 11 Ages x 8 Periods x 18 Cohorts.

**Age-Period-Cohort Models’ Constraints**

From the data in Table 1, 18 10-year birth cohorts can be calculated as linear outcomes of Period-Age = Cohort. The mortality rates can be analyzed with a general APC model specified as:

$\text{log} E\left( r_{ij} \right)=\text{log }E\left( \frac{d_{ij}}{n_{ij}} \right)=\beta_{0}+\beta_{i}^{A}+\beta_{j}^{P}+\beta_{k}^{C},$ (1)

where $\text{log} E\left( r_{ij} \right)$ is the logarithm of the expected heart disease mortality rate based on $d_{ij}$ deaths and $n_{ij}$ person-years in cell *ij* of a cross-classification of deaths and person-years in age interval *i* (for *i* = 1, . . ., *I* age groups) and time period *j* (for *j* = 1, . . . , *J* periods). Age and period effects are denoted by $\beta_{i}^{A}$ and $\beta_{j}^{P}$, respectively. $\beta_{k}^{C}$ denotes the *k*th (diagonal) birth cohort effect (for *k* = 1, …, *I + J*-1 birth cohorts), where the index *k* = *I* – *i* + *j*. For these data, *I* = 11 and *J* = 8 for *N* = *I* x *J* = 88 age x period cells covering 18 birth cohorts.

*CGLIM Constraint*

Kramer et al. (2015) used constrained generalized linear models (CGLIM) to explore period- and cohort-based variation in relative black-white differences in U.S. heart disease mortality. This “coefficients-constraints approach” (Yang and Land 2013b) places an equality constraint on one or more parameter vectors to yield unique age, period, and cohort effects. Kramer et al. (2015) constrained the effects of the first two period groups (1973-1977 and 1978-1982) to be equal such that $\beta_{1}^{P}-\beta_{2}^{P}=0.$ To test the within-method variability of the “coefficients-constraints approach,” we refit the CGLIM and constrained the mortality effects of the last two period groups (2003-2007 and 2008-2010) to be equal such that $\beta_{7}^{P}-\beta_{8}^{P}=0$. We also fit additional models that constrained the two middle time periods (1988-1992 and 1993-1997), constrained the first two (1888-1892 and 1893-1897) birth cohorts, and constrained and the last two birth cohorts (1968-1972 and 1973-1977).

*Intrinsic Estimator Constraint*

From equation (1), an ANOVA, centered-effects normalization is imposed on the effects $\sum\beta_{i}^{A}=\sum\beta_{j}^{P}=\sum\beta_{k}^{C}=0$, which can cast the terms:

$E\left( r_{ij} \right)=\tau_{0}\tau_{i}^{A}\tau_{j}^{P}\tau_{k}^{C},$ (2)

where the $\tau$ parameters under the APC model are multiplicative effects whose product is 1 over the levels of each factor. Under this normalization, the constant term in the model ($\tau_{0}$) is the scaled grand mean mortality rate from heart disease. The APC estimates thusly reflect the age, birth cohort, and time period deviations from the grand mean mortality rate for each population, net of the other APC effects. Early practitioners of this reparameterized model recognized the advantages of effect coding in dealing with the constraint (Kupper and Janis 1980), and the coding of the model design matrix became standard in development of the IE (Fu 2000, 2016; Yang and Land 2013a). Proponents of the IE advance a Moore-Penrose or generalized inverse of the less-than full-rank design matrix to provide a solution to the APC identification problem. The formal definitions of the IE and its properties as a statistical estimator are described in detail elsewhere (e.g., see Fosse and Winship 2018; Fu 2016; Powers 2014; Yang et al. 2008; Yang and Land 2013a, 2013b). Under the ANOVA type constraints, an omitted category must still be chosen to identify the model. A Stata program apc_ie (Schulhofer-Wohl and Yang 2006) available for fitting APC models using the IE omits the last APC categories by default, but a more flexible program ie_rate and ie_reg (Powers 2012) offers more possibilities for fitting APC models using the IE constraint under alternative specifications (StataCorp 2017). To test the within-method variability of the *IE approach* to estimating APC effects on black and white heart disease mortality, we use ie_rate (on mortality rates) and ie_reg (on mortality rate ratios) with the companion ie_norm utility to obtain the estimates of all the APC parameters (i.e., those estimated by ie_rate and ie_reg in addition to those pertaining to the omitted categories). We fit one APC model using the IE constraint that omits the last APC categories and fit a second APC model using the IE constraint that omits the first APC categories.

*Set Identification with a Maximum Entropy Constraint*

Browning et al. (2012: 5) express the APC coefficient vectors $\beta_{i}^{A}$, $\beta_{j}^{P}$, $\beta_{k}^{C}$ in terms of probability distributions over the set of “possible coefficient vectors.” The method chooses the probability distribution that best reflects the level of uncertainty in the available data (i.e., “choosing the most uninformative distribution possible” (11)). A Stata program apc (O’Dea 2012) uses set identification with maximum entropy by selecting the option ,method(me) and specifying the lower and upper bounds of the set of possible solutions, l() and u() (StataCorp 2017). The age-specific mortality rates in Table 1 are used to inform the bounds of the possible solutions using this maximum entropy estimator (MEE) on the mortality rates, and the rate ratios estimated between black and white women’s mortality rates and between black and white men’s mortality rates are used to inform the bounds of possible solutions on the rate ratios.

*HAPC Constraint using Markov Chain Monte Carlo simulations*

Bayesian statistical inference with a hierarchical APC (HAPC) model is used based on restricted maximum likelihood-empirical Bayes estimators through Monte Carlo simulations (Yang 2006; Yang and Land 2013b). The general HAPC approach nests fixed age effects in two higher-level units of cohorts and time periods estimated as random effects, which are often cross-classified in various combinations of time periods and birth cohorts (i.e., a cross-classified random effects model, CCREM). These models are usually fitted to individual-level data obtained from repeated cross-sectional surveys because these data structures nest individuals in cross-classifications of birth cohorts and time periods (Yang and Land 2013b). That is, survey respondents in the same age group observed during the same time period range need not be from the same birth cohort group, resulting in APC combinations that break the perfect linear dependency that exists in tabular rate data (i.e., Cohort $\neq$ Period-Age). However, this method can estimate APC effects in tabular rate data, but the added uncertainty due to the perfectly linear dependency (i.e., Cohort=Period-Age) in these data designs motivates the investigation of a full Bayesian alternative via the Bayes-HAPC.

The Level-1 model is specified as:

$R_{ijk}=\beta_{0jk}+\beta_{i}^{A}+e_{ijk}, e_{ijk}\sim N\left( 0,\sigma^{2} \right)$ (3)

And the Level-2 model is specified as:

$\beta_{0jk}=\gamma_{0}+\mu_{0j}+\upsilon_{0k}, \mu_{0j}\sim N\left( 0,\tau_{\mu} \right), \upsilon_{0k}\sim N\left( 0,\tau_{\upsilon} \right)$ (4)

In the combined model, $\beta_{0jk}$ is the “cell mean” log mortality rate for individuals in birth cohort *k* and time period *j.* The $\gamma_{0}$ is the model intercept, which is the expected value of $\beta_{0jk}$ for the omitted age interval *i* (for *i* = 1, . . ., *I* age groups).$\mu_{0j}$ is the random effect of time period *j* (i.e., the contribution of period *j* averaged across all birth cohorts) and is assumed to be normally distributed with mean 0 and variance $\tau_{\mu}$.$\upsilon_{0k}$ is the random effect of birth cohort *k* (i.e., the contribution of birth cohort *k* averaged across all time periods) and is assumed to be normally distributed with mean 0 and variance $\tau_{\upsilon}.$

The model uses restricted maximum likelihood-empirical Bayes (REML-EB) estimation, which estimates values of $\tau_{\mu}, \tau_{\upsilon},$and $\sigma^{2}$ that maximize the joint likelihood of these parameters given $R_{ijk}$ (i.e., observed log rates). To account for added uncertainty in these estimates, Markov chain Monte Carlo (MCMC) methods with Gibbs sampling were used to estimate four separate chains of 200,000 simulations with 150,000 burn-in simulations in OpenBUGS called from R using the BRugs package (R Core Team 2018; Thomas et al. 2006). Initial values for fixed effects age coefficients were set from a HAPC model estimated using lm in R. For APC models fitted to U.S. white men’s log heart disease mortality rates, we used noninformative inverse-gamma priors on $\sigma^{2}$ (.005, 1E-6), noninformative inverse-gamma priors on $\tau_{\mu}$ (.01, .01), and informative inverse-gamma priors on $\tau_{\upsilon}$ (2, 2.5) because Kramer et al. (2015) reported large cohort-based variation in U.S. heart disease mortality. For APC models fitted to log rate ratios between U.S. black and white men and U.S. black and white women we used noninformative inverse-gamma priors on $\sigma^{2}$ (.005, 1E-6), noninformative inverse-gamma priors on $\tau_{\mu}$ (.01, .01), and weakly informative inverse-gamma priors on $\tau_{\upsilon}$ (.5, .5). Examples of Stata and R code and simulation results from OpenBUGS are available below.

*Generalized Bayesian Age-Period-Cohort Model*

Age-specific heart disease mortality rates in Table 1 were used to estimate counts of heart disease deaths among U.S. white men in five-year age groups 35-39, …, 85+ for time periods 1973-1977, …, 2008-2010 by combining the Table 1 rates with population counts of U.S. white men from SEER (SEER 2018).

From equation (2), the sum-to-zero restrictions on all main effects are used. For the constant term in the model ($\tau_{0}$), a flat prior is used and the Bayesian APC model assumes that the first differences of age, period, and cohort parameters are independent Gaussian random variables. For example, for the period effects, $\tau^{P}$, a smoothing prior based on first differences is given by

$p\left( \tau^{P}|\lambda\right)\propto\text{exp}\left( -\frac{\lambda}{2}\sum_{j=2}^{J} \left( \tau_{j}^{P}-\tau_{j-1}^{P} \right)^{2} \right),$

where $\lambda$ is a precision parameter that determines the smoothness of the estimated period effects. The $\lambda$ prior corresponds to the formulation as a *first-order random walk* (RW1) $\tau_{j}^{P}$~ *N*($\tau_{j-1}^{P},\lambda^{-1}$), *j* = 2, …, *J*. The RW1 model approaches the identifiability problem by imposing a stochastic constraint in which the RW1 model will prefer the transformations of the age, period, and cohort in equation (2) that minimize the quadratic first differences. The uncertainty about the precision parameters for $\tau_{i}^{A}, \tau_{j}^{P}, \tau_{k}^{C}$ (*k*, $\lambda$, and $\nu$) is directly incorporated in the estimation of $\tau_{i}^{A}, \tau_{j}^{P}, \tau_{k}^{C}$.

The default flat priors in the bamp R package were used for the age, period, and cohort parameters (.0005) and the model was extended to include account for overdispersion (i.e., unstructured heterogeneity) in the counts of heart disease-related deaths (.05). We used the bamp package with 150,000 iterations and 30,000 burn-ins. Examples of R code and are available below and see Knorr-Held and Rainier (2001) and Schmid and Held (2004) for expanded specifications and discussions of generalized Bayesian APC models on mortality trend analyses.

**Supplementary Figure 1**. Relative Ratios Between U.S. Black and White Men’s and Women’s Age-specific Heart Disease Mortality Rates, 1973-2010.

Note: Darker shades indicate younger ages and lighter shades indicate older ages.

**Supplementary Figure 2**. Relative Ratios Between U.S. Black and White Men’s and Women’s Age Standardized Heart Disease Mortality Rates, 1973-2010.

Stata Scripts used to fit APC IE, APC CGLIM, and APC MEE for U.S. white men’s heart disease mortality rates.

*************

* White Men *

*************

use "/…/white men.dta", clear

drop if period == .

label define agel 1 "35-39" 2 "40-44" 3 "45-49" 4 "50-54" 5 "55-59" ///

6 " 60-64" 7 "65-69" 8 "70-74" 9 "75-79" 10 "80-84" 11 "85+"

label values age agel

replace period = period+11

gen cohort = period - age

apc_ie rate, age(age) period(period) cohort(cohort) family(poisson) link(log) scale(dev) irls

pause

/* ANOVA Normalization */

/* Last Category Levels as Reference */

qui tab age, gen(a_)

scal arow = r(r)

qui tab period, gen(p_)

scal prow = r(r)

qui tab cohort, gen(c_)

scal crow = r(r)

* construct ANOVA normalization

forval i = 1/`=arow' {

gen aC`i' = a_`i' - a_`=arow'

}

forval i = 1/`=prow' {

gen pC`i' = p_`i' - p_`=prow'

}

forval i = 1/`=crow' {

gen cC`i' = c_`i' - c_`=crow'

}

ie_rate rate aC1-aC10 pC1-pC7 cC1-cC17

mata

b = st_matrix("e(b)")

V = st_matrix("e(V)")

bAref = -sum(b[1]..b[10])

sebAref = sqrt(sum(V[1..10,1..10]))

bPref = -sum(b[11..17])

sebPref = sqrt(sum(V[11..17,11..17]))

bCref = -sum(b[18..34])

sebCref = sqrt(sum(V[18..34,18..34]))

("Estimates and Std. Errors")

(bAref, sebAref)

(bPref, sebPref)

(bCref, sebCref)

end

ie_norm, groups(aC1-aC11, pC1-pC8, cC1-cC18)

estat ic

/* ANOVA Normalization*/

/* First Category Levels as References */

drop aC* pC* cC* a_* p_* c_*

qui tab age, gen(a_)

scal arow = r(r)

qui tab period, gen(p_)

scal prow = r(r)

qui tab cohort, gen(c_)

scal crow = r(r)

* construct ANOVA normalization

forval i = 1/`=arow' {

gen aCF`i' = a_`i' - a_1

}

forval i = 1/`=prow' {

gen pCF`i' = p_`i' - p_1

}

forval i = 1/`=crow' {

gen cCF`i' = c_`i' - c_1

}

ie_rate rate aCF2-aCF11 pCF2-pCF8 cCF2-cCF18

mata

b = st_matrix("e(b)")

V = st_matrix("e(V)")

bAref = -sum(b[1]..b[10])

sebAref = sqrt(sum(V[1..10,1..10]))

bPref = -sum(b[11..17])

sebPref = sqrt(sum(V[11..17,11..17]))

bCref = -sum(b[18..34])

sebCref = sqrt(sum(V[18..34,18..34]))

("Estimates and Std. Errors")

(bAref, sebAref)

(bPref, sebPref)

(bCref, sebCref)

end

ie_norm, groups(aCF1-aCF11, pCF1-pCF8, cCF1-cCF18)

estat ic

* Constrained

tab age, gen(agec)

tab period, gen(perc)

tab cohort, gen(cohc)

* Kramer

glm rate agec1 agec2 agec3 agec4 agec5 agec6 agec8 agec9 agec10 agec11 ///

perc3 perc4 perc5 perc6 perc7 perc8 cohc1 cohc2 cohc3 cohc4 cohc5 cohc6 ///

cohc7 cohc8 cohc9 cohc10 cohc12 cohc13 cohc14 cohc15 cohc16 cohc17 cohc18, ///

link(log) family(poisson)

* Middle Periods

glm rate agec1 agec2 agec3 agec4 agec5 agec6 agec8 agec9 agec10 agec11 ///

perc1 perc2 perc3 perc6 perc7 perc8 cohc1 cohc2 cohc3 cohc4 cohc5 cohc6 ///

cohc7 cohc8 cohc9 cohc10 cohc12 cohc13 cohc14 cohc15 cohc16 cohc17 cohc18, ///

link(log) family(poisson)

* Constrain First Cohorts

glm rate agec1 agec2 agec3 agec4 agec5 agec6 agec8 agec9 agec10 agec11 ///

perc1 perc2 perc3 perc5 perc6 perc7 perc8 cohc3 cohc4 cohc5 cohc6 ///

cohc7 cohc8 cohc9 cohc10 cohc11 cohc12 cohc13 cohc14 cohc15 cohc16 cohc17 cohc18, ///

link(log) family(poisson)

* Constrain Middle Cohorts

glm rate agec1 agec2 agec3 agec4 agec5 agec6 agec8 agec9 agec10 agec11 ///

perc1 perc2 perc3 perc5 perc6 perc7 perc8 cohc1 cohc2 cohc3 cohc4 cohc5 cohc6 ///

cohc7 cohc10 cohc11 cohc12 cohc13 cohc14 cohc15 cohc16 cohc17 cohc18, ///

link(log) family(poisson)

* Constrain Last Cohorts

glm rate agec1 agec2 agec3 agec4 agec5 agec6 agec8 agec9 agec10 agec11 ///

perc1 perc2 perc3 perc5 perc6 perc7 perc8 cohc1 cohc2 cohc3 cohc4 cohc5 cohc6 ///

cohc7 cohc8 cohc9 cohc10 cohc11 cohc12 cohc13 cohc14 cohc15 cohc16, ///

link(log) family(poisson)

* APC on rate - Poisson Model

apc_ie rate, age(age) period(period) cohort(cohort) family(poisson) link(log) scale(dev) irls

pause

* APC on lograte - Regression, to be consistent with Max Entropy Model

gen lograte = log(rate)

apc_ie lograte, age(age) period(period) cohort(cohort)

pause

* Maximum Entropy

apc lograte, age(age) period(period) cohort(cohort) method(me) l(2.04) u(8.6)

pause

R Scripts used to fit HAPC-CCREM-MCMCs for U.S. white men’s heart disease mortality rates.

# R Analyses of White Men NVSS Circ Disease Mortality

library(foreign)

statad1 <- read.dta("…/wm_data.dta")

statad1 [1,]

period <-statad1$period

age <-statad1$age

cohort <-statad1$cohort

y <- statad1$rate

age1 <- statad1$agec1

age2 <- statad1$agec2

age3 <- statad1$agec3

age4 <- statad1$agec4

age5 <- statad1$agec5

age6 <- statad1$agec6

age7 <- statad1$agec7

age8 <- statad1$agec8

age9 <- statad1$agec9

age10 <- statad1$agec10

age11 <- statad1$agec11

library(lme4)

library(BRugs)

library(R2OpenBUGS)

library(coda)

library(MASS)

logy <- log(y)

m1 <- lmer(logy ~ age1+age2+age3+age4+age5+age6+ age8+age9+age10+age11 + (1|period) + (1|cohort))

# extract RE

R<- ranef(m1)

U <- R$period

V <- R$cohort

R

m1

M <- length(unique(period))

perID <- unique(period)

c1 <- as.numeric(cohort==1)

c2 <- as.numeric(cohort==2)

c3 <- as.numeric(cohort==3)

c4 <- as.numeric(cohort==4)

c5 <- as.numeric(cohort==5)

c6 <- as.numeric(cohort==6)

c7 <- as.numeric(cohort==7)

c8 <- as.numeric(cohort==8)

c9 <- as.numeric(cohort==9)

c10 <- as.numeric(cohort==10)

c11 <- as.numeric(cohort==11)

c12 <- as.numeric(cohort==12)

c13 <- as.numeric(cohort==13)

c14 <- as.numeric(cohort==14)

c15 <- as.numeric(cohort==15)

c16 <- as.numeric(cohort==16)

c17 <- as.numeric(cohort==17)

c18 <- as.numeric(cohort==18)

p1 <- as.numeric(period==12)

p2 <- as.numeric(period==13)

p3 <- as.numeric(period==14)

p4 <- as.numeric(period==15)

p5 <- as.numeric(period==16)

p6 <- as.numeric(period==17)

p7 <- as.numeric(period==18)

p8 <- as.numeric(period==19)

b.mle <- coef(lm(logy ~ age1+age2+age3+age4+age5+age6+ age8+age9+age10+age11 + p2+p3+p4+p5+p6+p7+p8+ c1+c2+c3+c4+c5+c6+c7+c8+c9+c10+ c12+c13+c14+c15+c16+c17+c18))

b.mle

N <- length(logy)

A <- array(c(age1,age2,age3,age4,age5,age6,age8,age9,age10,age11), c(88,10))

perID <- period-11

cohID <- cohort

# data for MCMC

data <- list(N=N,

M=M,

Y=logy,

perID=perID,

cohID=cohID,

A=structure(.Data=A, .Dim=c(88,10))

)

#MCMC model

APCmod<- function()

{

# normal priors on random effects

for ( k in 1 : M) {

U[k] ~ dnorm(0, tauU)

}

for ( j in 1: 18) {

V[j] ~ dnorm(0,tauV)

}

# likelihood Y~dnorm(mu)

for ( i in 1 : N ) {

Y[i] ~ dnorm(mu[i], tau.y)

mu[i] <- a + inprod(b[],A[i,]) + U[perID[i]] + V[cohID[i]]

}

tau.y ~ dgamma(.005, 1.0E-6)

sigma.y <- 1/sqrt(tau.y)

# normal priors on fixed effect (with big variances so as to make them uninfomative)

a ~ dnorm(0,1.0E-6)

for(i in 1:10) {

b[i] ~ dnorm(0,1.0E-6)

}

# informative priors on variance components

tauU ~ dgamma(.01, .01)

tauV ~ dgamma(2, 2.5)

# transform precisions to variances

sigma2U <- 1/tauU

sigma2V <- 1/tauV

}

## some temporary filename:

filename <- file.path("…", "APCmod.txt")

## write model file: writeModel (BRugs) vs. write.model (R2WinBUGS)

writeModel(APCmod, filename)

## and let's take a look:

file.show(filename)

inits1 <- function() { list(a=b.mle[1], b=b.mle[2:11], tauU=1/.02, tauV=1/.2)

}

# update inside of OpenBUGS

bugs(data, inits=inits1, debug=TRUE , model.file="/…/APCmod.txt",

n.chains=4, parameters = c("a","b", "sigma2U", "sigma2V", "U", "V", "sigma.y", "tau.y"), n.iter=300000, n.burnin=100000, n.thin=1)

Sample of results from HAPC-CCREM-MCMC fitted to U.S. white men’s heart disease mortality rates.


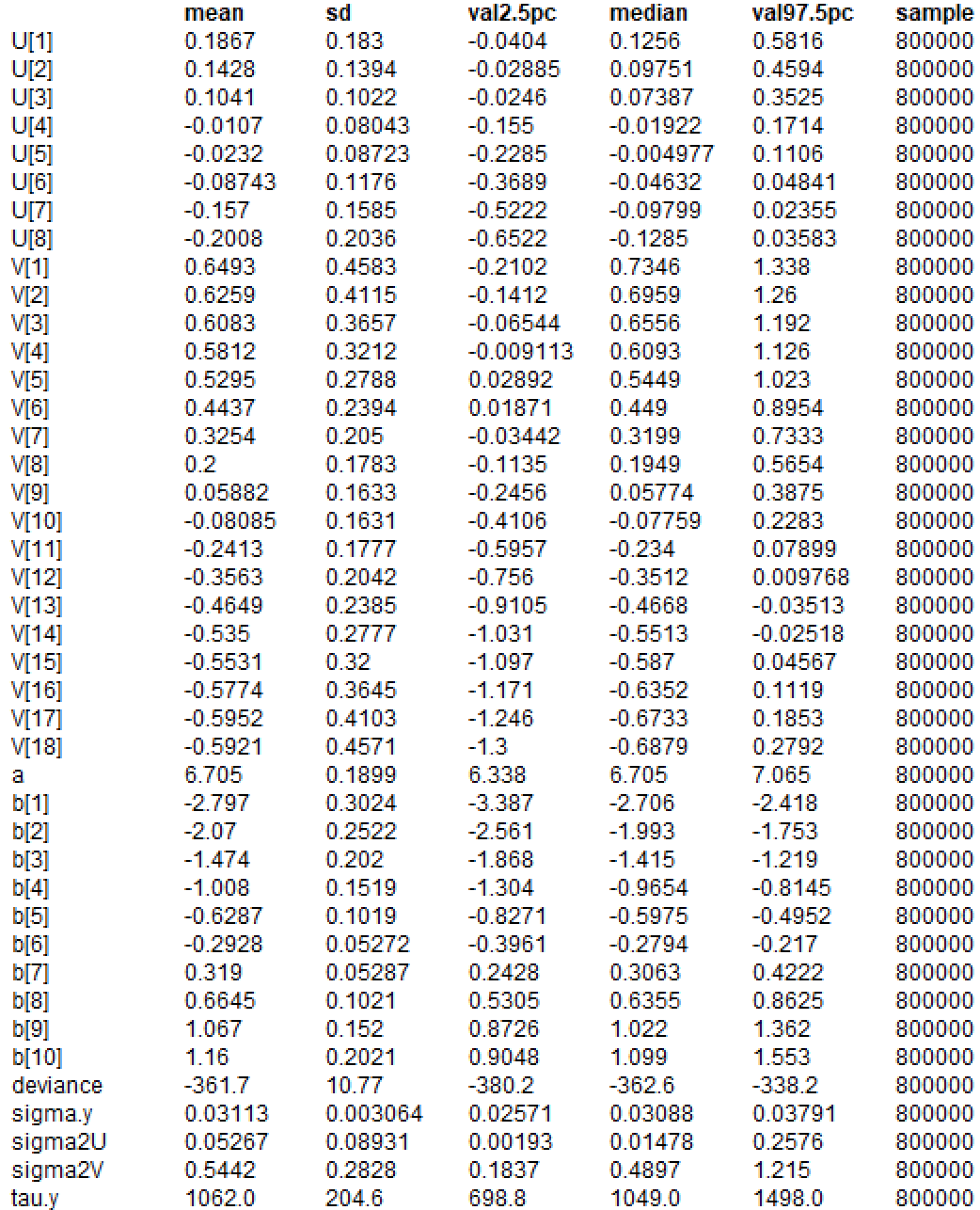


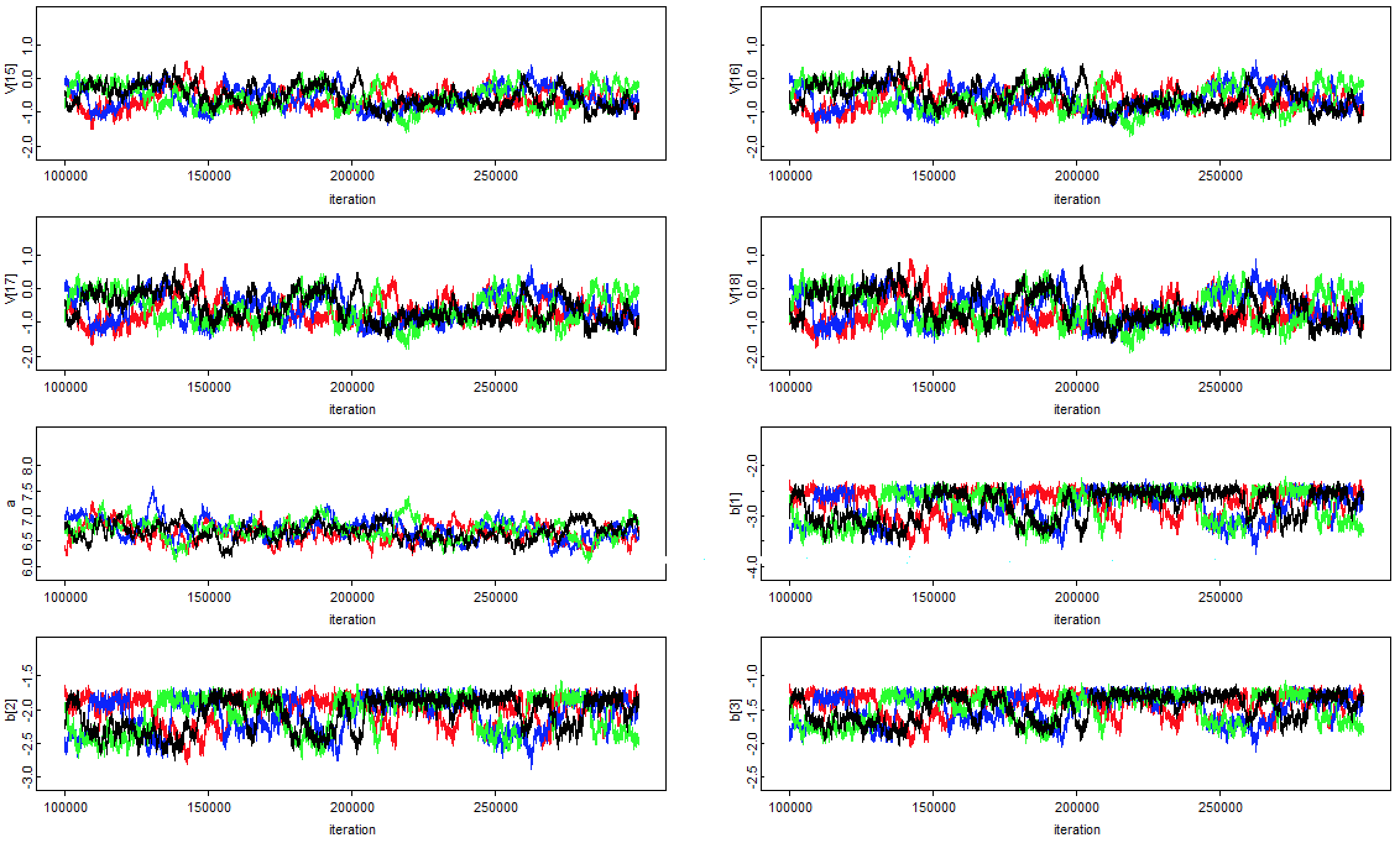


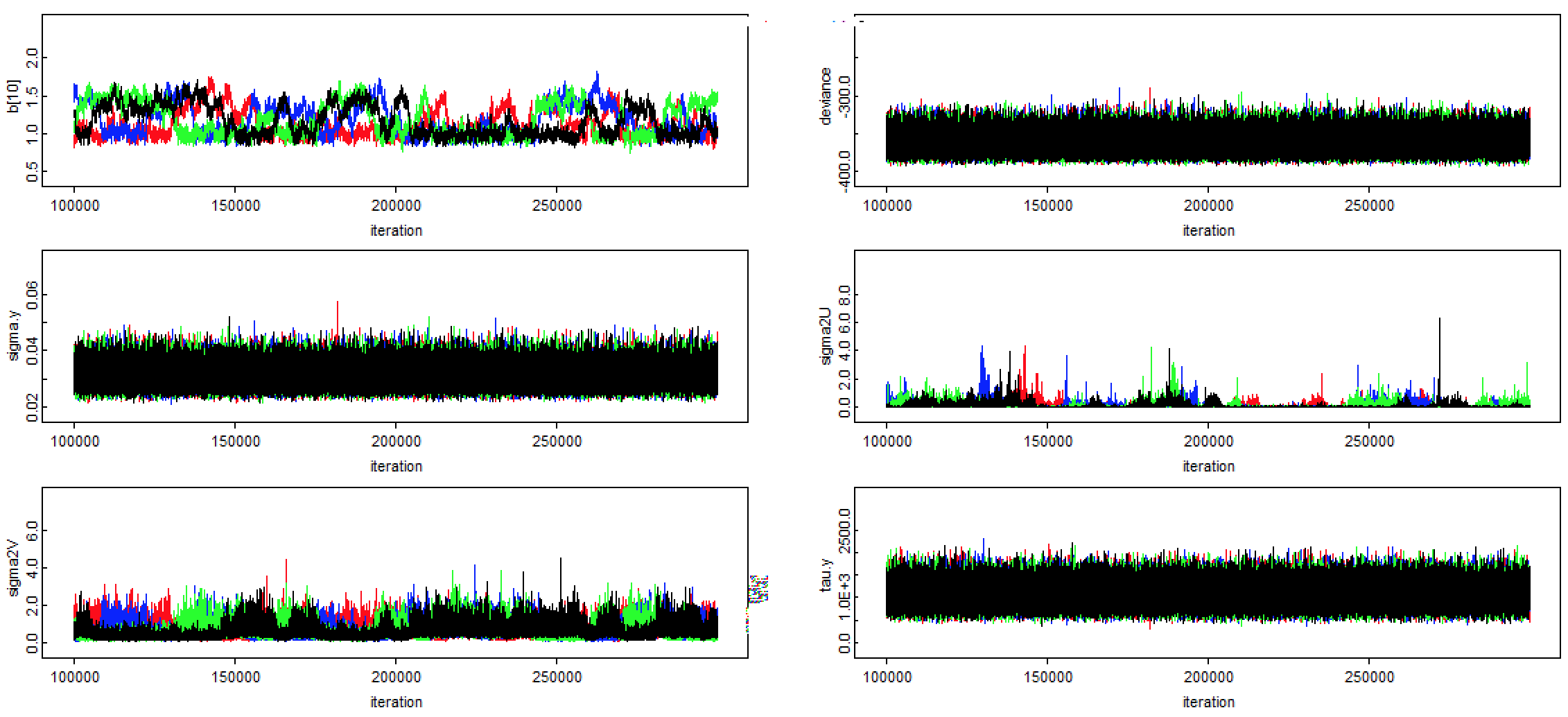


R Scripts used to fit Bayesian APC Models to U.S. white men’s heart disease mortality.

### White Men, Heart Disease Mortality ##

### Bayesian APC by bamp (https://volkerschmid.github.io/bamp/) in R ##

# read the population counts

# official U.S. population counts, 1973-2010

pop <- data.matrix(read.table("/Users/ryan/Documents/Papers/APC Guidelines/Submission/PlosOne/R&R/white_men_pop.txt"))

# read the HD death counts

# estimated using Kramer et al. Table 1 and age-specific X period-specific population counts

heart <- data.matrix(read.table("/Users/ryan/Documents/Papers/APC Guidelines/Submission/PlosOne/R&R/white_men_cases.txt"))

dimnames(pop) <- NULL

dimnames(heart) <- NULL

pop

heart

library(bamp)

# BAMP Model - 1 Age X Period for linear dependency and overdispersion

# First-order random walks as smoothing parameters for age, period, and cohort parameters

# 30K burn-in models

# 120K additional iterations

# Store every 50th iteration

# Weak hyperparameters (default settings used)

### a=1, b=.0005 for APC effects, b=.05 for overdispersion

model1 <- bamp(heart, pop, age="rw1", period="rw1", cohort="rw1", overdisp=TRUE, periods_per_agegroup = 1, mcmc.options=list(number_of_iterations=150000, burn_in=30000, step=50, tuning=500))

model1

checkConvergence(model1)

effects(model1)
