# Supplementary material for: Clarifying assumptions in age-period-cohort analyses and validating results
Source: PLoS One. 2020 Oct 6;15(10):e0238871. doi: 10.1371/journal.pone.0238871 (PMC7537862; doi:10.1371/journal.pone.0238871)
Supplement: Attachment — Submitted filename: Response to Review.docx [file pone.0238871.s002.docx]

Ms. No.: PONE-D-20-06144

Journal: *PLOS ONE*

We are delighted to receive an invitation to revise and resubmit our manuscript for possible publication in *PLOS ONE*. We thank the referees for their constructive comments and suggestions. Below we have quoted reviewer comments, followed by our responses.

**Referee 1**

**Overall, the paper is reasonably well written, the analyses are clearly described (with data that can be used for independent verification), and the conclusions are well taken therefrom.

Here are a couple of things to attend to in a revision:

In the Data section, you state: “From the data in Table 1, 18 10-year birth cohorts can be calculated as linear outcomes of Period-Age = Cohort.” First, do you mean 5-year birth cohorts, as the data in Table 1 are in 5-year age and time period groupings? Second, it would be good to indicate how the number of cohorts is calculated from the table.**

We apologize for not making clear the data structure in the manuscript. As the design matrix is vitally important for some APC models’ estimates, this was an oversight and we thank the Referee for spotlighting the error. We now clarify the Age x Period structure (11 x 8) and the resulting 18 10-year birth cohorts. We also now include a table showing the design of the data structure in the appendix. For convenience, please see the structure below (although it may be too small to see the details), wherein we highlight the five-year age groups by alternating blue and white rows and highlight the five-year period groups (and one three-year period, 2008-2010) by alternative yellow and white columns. Select 10-year cohorts are highlighted in different shades of green.

**It might be meritorious to include a brief statement on the concept of estimable functions in CGLIM APC models, how this has played a key role in the APC body of statistical methodology literature (going back to Kupper et al. 1985), how a statistical test (in Yang et al. 2008 and Yang and Land 2013) can be applied to CGLIM estimates (such as those in your various figures) of the A, P, and C coefficients to assess the extent to which they are within sampling error of the Intrinsic Estimator (which does satisfy the Kupper et al. condition).**

This is a good point and we thank the referee for suggesting it. We don’t commit the space to these important considerations, but we now refer the reader to some existing works that do well to discuss the history of these estimators (e.g., Land and Yang 2013; Land et al. 2016; Yang et al. 2004). We are also aware of excellent work (not yet published, but likely available soon) that more fully attends to these matters.

**Referee 2**

**A practical guideline for how to adequately apply the best tools for a given topic and dataset and how to adjudicate among existing solutions is sorely needed. This paper does just that! I have seen a handful of papers that have attempted at this goal, but none has done as well and thorough a job as this one.**

We thank the Referee for their enthusiasm for the manuscript and their supportive comments.

**A small suggestion: p.6., 1.c the term “researcher involvement” is a bit vague and can be more clearly stated as something like “subjective choice” or “arbitrary constraint”.**

This is a helpful suggestion. After rereading the manuscript, we agree that our continued use of “researcher involvement” in many places is vague and “subjective choice” and “arbitrary constraint” are better options. Also, we now cite O’Brien (2015) and Fosse and Winship (2019) and adopt their use of “explicit” constraints (e.g., equality constraints) and “mechanical” constraints (e.g., the intrinsic estimator’s constraint on the design matrix) to help clarify the distinctions between models’ various constraints.

**Referee 3**

**The goal of this paper is to overcome the "more heat than light" problem resulting from the debate about APC methods in social and medical sciences… In general, I find that this is a valuable contribution. I like the idea of the hypothetical 8 research teams. I completely agree with the authors' suggestions 1 and 3.**

**I have a couple of fairly minor concerns about the paper. The first is that I don't this suggestion #2 is strictly necessary. In most cases, there is literally no difference between estimating (say) two separate models for men and women and estimating a model where all covariates are interacted with sex. I realize that for many models (like the HAPC-CCREM) doing this properly would involve creating separate period and cohort variable values for men and women so that the random intercepts are estimated separately. Many researchers probably would not do this (or think of this). So, as a practical matter, perhaps the authors' suggestion is reasonable. But it might be worth noting that there are alternatives**.

We thank the Referee for their supportive comments and also for their perceptive and correct comments about the non-differences between APC estimates from the pooled model with interactions and APC estimates from models fitted separately to the subpopulations. We have added a sentence stating this fact, but continue to advocate for point 1.b. for readers’ benefits (e.g., clarity and ease of verifying the assumptions).

**The second (minor) concern is whether certain methods are more likely to agree with each other in general, making "votes" between approaches not quite independent. I am very familiar with HAPC-CCREMs and CGLIMs because I work in a field that almost always uses individual data rather than tables. So I'm less familiar with (say) the IE. But if methods have similar assumptions might they not tend to agree even if they are "wrong"? That may be worth mentioning.**

This is an excellent point. Yes, many APC models share underlying estimators and assumptions and whose “mechanical” constraints are quite similar to each other. The shared properties of different estimators likely increase the likelihood that these models will estimate similar APC parameters, which might provide false reassurance b/c all models could be biased. We cite O’Brien (2011, 2015) about the perpendicular nature of constrained estimators, Fu (2016) about the data dependency of constrained estimators, and Fosse and Winship (2018) about the shared properties and differences of Moore-Penrose Estimators of APC effects. Land et al. (2016) have also highlighted works that discuss shared properties of estimators: “the asymptotic convergence of APC estimators to the IE has been studied (Fu and Hall [2006](https://www.journals.uchicago.edu/doi/full/10.1086/689853?casa_token=Rmz2Ac2jJ4QAAAAA%3A2NfA7L67nsrfce6RN5sQy8PLh0pibapge_2LbzpLQ1x33VLYkM0CnQiOrVrlhf6Z-i9QcmuuH9XL&#rf4); Fu [2008](https://www.journals.uchicago.edu/doi/full/10.1086/689853?casa_token=Rmz2Ac2jJ4QAAAAA%3A2NfA7L67nsrfce6RN5sQy8PLh0pibapge_2LbzpLQ1x33VLYkM0CnQiOrVrlhf6Z-i9QcmuuH9XL&#rf3)), and Xu and Powers ([2015](https://www.journals.uchicago.edu/doi/full/10.1086/689853?casa_token=Rmz2Ac2jJ4QAAAAA%3A2NfA7L67nsrfce6RN5sQy8PLh0pibapge_2LbzpLQ1x33VLYkM0CnQiOrVrlhf6Z-i9QcmuuH9XL&#rf17)) showed that a Bayesian ridge model with a common prior for the ridge parameter yields estimates of age, period, and cohort effects similar to those based on the IE and to those based on a ridge estimator”(967).

Fosse and Winship discuss at length the differences between MP estimators, which lead them to ultimately advocate the “orthogonal estimator” (OE) over other alternatives:

MP estimators differ from one another in a vitally important way: Because they are based on different design matrices, and because different design matrices change the location of the origin relative to the solution line, they will produce quite different estimates. In general the estimates of the linear effects produced by an MP estimator, including the IE, will vary depending on at least three aspects of the data: first, the number of APC groups in the data set, e.g., the number of periods; second, the size and sign of the nonlinearities; and third, the choice of the reference category. This lack of robustness is a serious weakness.

However, as it’s long been known (e.g., see Kupper and Janis 1985), the differences between the estimators (i.e., “lack of robustness”) are minimized as the design matrix increases. So, the referee raises a good point that 1) APC models using similar estimators (e.g., MP, ridge estimators, principal components, etc.) and 2) fitted to large design matrices will likely estimate similar APC coefficients.

While the HAPC-CCREM does not share an underlying estimator with either the IE or with set identification using maximum entropy estimator, the latter two are both shrinkage estimators/Moore-Penrose and therefore may be likely to converge on similar APC estimates. We now provide additional evidence for applying guideline point #3 (i.e., test between-method consistency) by providing estimates from a Bayesian Age-Period-Cohort Model (Knorr-Held and Rainer 2001; Schmid 2020) using the bamp package in R. We did so because this approach uses a “mechanical” constraint via first-order random walks as smoothing parameters, an overdispersion term, and statistical inference by Markov chain Monte Carlo (MCMC) methods. The data and analytic scripts used to fit these models in R are provided in the appendix.

The normalized APC estimates from the Bayesian APC Model are provided in Figure 3 and are very similar to estimates from the IE, MEE, and HAPC-CCREM. However, the model is fitted to counts of death assuming a Poisson distribution and using log link (as opposed to modeling the rates or rate ratios between the population) so we did not use Bayesian APC Models to estimate black-white differences in heart disease mortality rates.

**…this paper is a very valuable practical contribution and solid advice. Researchers using any techniques should always check for robustness of their results to alternative reasonable specifications. This set of guidelines should help bring this into being in the APC world.**

We thank the Referee for their supportive comments about the paper’s contribution.

**Referee 4**


**I do not find any technical problems with the paper, but I also don’t find anything new here. There have been a number of papers over the years that have offered suggestions for appropriate use of APC methods, including some of the papers cited by the authors. The suggestions discussed and demonstrated here have each been discussed in other papers. It’s not clear that this paper makes a significant contribution to the literature.**

It is true that many existing papers have offered suggestions and solutions to the APC conundrum. Most works, however, have advanced one technique over others. For example, recent work by Fosse and Winship (2018) reviews similarities and differences between a set of Moore-Penrose Estimators for APC effects and concludes with a set of guidelines to adopt the “orthogonal estimator” over other alternatives (the IE included). This example is pretty standard for APC papers: review the conundrum, synthesize existing approaches (while highlighting their potential shortcomings), and propose a new technique for researchers to adopt.

In terms of the paper’s novelty and the usefulness of the guidelines therein, we agree with Referee 2’s summary of the literature and the usefulness of extant “guides”:

**The history of the cottage industry of APC analysis has been full of contentious debates over the validities of various methodological approaches to estimating the APC regression models and further identifying the meaningful or “true” temporal trends attributable to period or cohort effects… , the area of APC analysis has been plagued by confusions as a result of lack of understandings of the nature of the “model identification problem”, wrongful criticisms of existing and new methods arising from such misunderstandings, and consequently inadequate applications of analytic approaches (descriptive and multivariate). Suffice it to say, it is a such mess. The golden standard of good science, namely, replications of findings, has been elusive in this odd case. A practical guideline for how to adequately apply the best tools for a given topic and dataset and how to adjudicate among existing solutions is sorely needed.

This paper does just that! I have seen a handful of papers that have attempted at this goal, but none has done as well and thorough a job as this one. The problem with prior studies trying to defeat certain methodologies or recommending new ones is that they are charged with priori conclusions – they have decided what to reject or support prior to the actual algebraic development.**

To recap, the bulk of the APC literature proposes and advances new approaches, considerations, and/or estimators (often against the use of others). The piecemeal and fragmented literature is likely partly responsible for the various APC estimates that allows for such strong disagreements and fuels Harper’s (2015:2) perception, “it seems likely that researchers could end up choosing APC models that are most consistent with their favorite hypotheses.” Here, rather than propose a new technique (or some variant of an existing estimator) against existing solutions to the APC conundrum, we layout a practical guide for researchers in the hopes that following such standard practices will reduce the likelihood of different APC analyses arriving at different results.

**I would add that I don’t find APC analyses in general to be particularly useful, because they don’t answer a theory-driven question. Instead, one is left without an explanation for exactly what produced period or cohort effects. It is more advisable, rather than adopting yet another set of standards for conducting these atheoretical analyses, to simply include the cohort or period factors into the model that produce the effects. That approach doesn’t require arbitrary decisions to break linear dependence of effects.**

While the history of cohort analyses – and perhaps the best and/or most appropriate use of APC models – is descriptive accounts of trends, APC models can be theoretically motivated. Disentangling period-based sources of variation in trends from cohort-based sources of variation in trends can be highly relevant to theories of social change, disease prevalence, or demographic processes. Further, the models can be used to empirically validate theoretical claims about sources of trends in health, economic, and social outcomes.

In this manuscript, we’re considering the underlying source of changes in black-white differences in heart disease mortality. Cohort-based sources (e.g., differences in historical experiences of early-life exposures, racial inequalities in access to and use of health-enhancing technologies, race differences in health risk behaviors [e.g., nutrition, stress, smoking]) and period-based sources of changes (e.g., policies or recent diffusion of medical technologies) imply very different theories about the drivers of U.S. mortality trends and how these drivers may be different for U.S. race/ethnic populations.
