# Supplementary material for: Clarifying assumptions in age-period-cohort analyses and validating results
Source: PLoS One. 2020 Oct 6;15(10):e0238871. doi: 10.1371/journal.pone.0238871 (PMC7537862; doi:10.1371/journal.pone.0238871)
Supplement: Peer review history

# Peer review: Decision Letter 0

**DOI:** 10.1371/journal.pone.0238871.r001

**Authors:** Bryan L. Sykes

21 May 2020

PONE-D-20-06144

Clarifying Assumptions in Age-Period-Cohort Analyses and Validating Results

PLOS ONE

Dear Dr. Masters,

Thank you for submitting your manuscript to PLOS ONE. After careful consideration, we feel that it has merit but does not fully meet PLOS ONE’s publication criteria as it currently stands. Therefore, we invite you to submit a revised version of the manuscript that addresses the points raised during the review process.

Your paper was reviewed by four leading scholars of APC methods, with the vast majority recognizing the novelty and importance of your research.  My read of your work is that it has the potential to clarify a significant amount of disagreement and confusion over when and how to execute APC methods and how to assess their corresponding results.  However, reviewers have identified five areas for revision before your paper can move forward in the publication process.

First, Reviewer 4 is concerned that your paper does not offer substantial guidance beyond the work you already cite (i.e., past recommendations on APC methods).  Please draw out your major contribution a bit more by explicating 1) how your recommendations resolve an existing debate in the American Journal of Epidemiology and 2) how your work advances a new (and clearer) set of guidelines for executing and assessing APC methods and results.

Second, Reviewer 3 would like for you to explain whether certain methods are more likely to agree in APC models and analyses.  This methodological inquiry would certainly draw out your contribution a bit more (in relation to Reviewer 4's concern).  Along these lines, Reviewer 2 also questions whether suggestion #2 is necessary.  Please provide a stronger justification for your second methodological suggestion, which would also bolster the contribution of your work.

Third, Reviewer 1 requests that you clarify how birth cohorts are calculated in the Data section of the paper, noting that Table 1 contains five-year age groups and periods, not "18 10 year birth cohorts."  Do you mean that a 5-year age group across a 5-year period corresponds to 10 1-year birth cohorts?  Either way, please clarify the meaning of this passage for readers.

Fourth, Reviewer 2 would like you to use different language (or word choices) to denote "researcher involvement."  The reviewer suggests, instead, that you refer to such terms as "subjective choice" or "arbitrary constraint".

Finally, please verify that all works cited in the manuscript are listed in the bibliography (as noted by Reviewer 1).  Along these lines, I believe it may be useful for you to cite, or engage with, the APC approaches of John Wilmoth (1990) and Wenjiang Fu (2008), published in Sociological Methodology and Sociological Methods & Research, respectively. 

Please submit your revised manuscript by Jul 05 2020 11:59PM. If you will need more time than this to complete your revisions, please reply to this message or contact the journal office at plosone@plos.org. When you're ready to submit your revision, log on to https://www.editorialmanager.com/pone/ and select the 'Submissions Needing Revision' folder to locate your manuscript file.

Please include the following items when submitting your revised manuscript:

- A rebuttal letter that responds to each point raised by the academic editor and reviewer(s). You should upload this letter as a separate file labeled 'Response to Reviewers'.
- A marked-up copy of your manuscript that highlights changes made to the original version. You should upload this as a separate file labeled 'Revised Manuscript with Track Changes'.
- An unmarked version of your revised paper without tracked changes. You should upload this as a separate file labeled 'Manuscript'.

If you would like to make changes to your financial disclosure, please include your updated statement in your cover letter. Guidelines for resubmitting your figure files are available below the reviewer comments at the end of this letter.

If applicable, we recommend that you deposit your laboratory protocols in protocols.io to enhance the reproducibility of your results. Protocols.io assigns your protocol its own identifier (DOI) so that it can be cited independently in the future. For instructions see: http://journals.plos.org/plosone/s/submission-guidelines#loc-laboratory-protocols

We look forward to receiving your revised manuscript.

Kind regards,

Bryan L. Sykes, Ph.D.

Academic Editor

PLOS ONE

Journal requirements:

When submitting your revision, we need you to address these additional requirements:

1.    Please ensure that your manuscript meets PLOS ONE's style requirements, including those for file naming. The PLOS ONE style templates can be found at http://www.plosone.org/attachments/PLOSOne_formatting_sample_main_body.pdf and http://www.plosone.org/attachments/PLOSOne_formatting_sample_title_authors_affiliations.pdf

2. Please include captions for your Supporting Information files at the end of your manuscript, and update any in-text citations to match accordingly. Please see our Supporting Information guidelines for more information: http://journals.plos.org/plosone/s/supporting-information.

[Note: HTML markup is below. Please do not edit.]

Reviewers' comments:

Reviewer's Responses to Questions

**Comments to the Author**

1. Is the manuscript technically sound, and do the data support the conclusions?

The manuscript must describe a technically sound piece of scientific research with data that supports the conclusions. Experiments must have been conducted rigorously, with appropriate controls, replication, and sample sizes. The conclusions must be drawn appropriately based on the data presented. 

Reviewer #1: Yes

Reviewer #2: Yes

Reviewer #3: Yes

Reviewer #4: Yes

**********

2. Has the statistical analysis been performed appropriately and rigorously? 

Reviewer #1: Yes

Reviewer #2: Yes

Reviewer #3: Yes

Reviewer #4: Yes

**********

3. Have the authors made all data underlying the findings in their manuscript fully available?

The PLOS Data policy requires authors to make all data underlying the findings described in their manuscript fully available without restriction, with rare exception (please refer to the Data Availability Statement in the manuscript PDF file). The data should be provided as part of the manuscript or its supporting information, or deposited to a public repository. For example, in addition to summary statistics, the data points behind means, medians and variance measures should be available. If there are restrictions on publicly sharing data—e.g. participant privacy or use of data from a third party—those must be specified.

Reviewer #1: Yes

Reviewer #2: Yes

Reviewer #3: Yes

Reviewer #4: Yes

**********

4. Is the manuscript presented in an intelligible fashion and written in standard English?

PLOS ONE does not copyedit accepted manuscripts, so the language in submitted articles must be clear, correct, and unambiguous. Any typographical or grammatical errors should be corrected at revision, so please note any specific errors here.

Reviewer #1: Yes

Reviewer #2: Yes

Reviewer #3: Yes

Reviewer #4: Yes

**********

5. Review Comments to the Author

Please use the space provided to explain your answers to the questions above. You may also include additional comments for the author, including concerns about dual publication, research ethics, or publication ethics. (Please upload your review as an attachment if it exceeds 20,000 characters)

Reviewer #1: This paper proposes three guidelines to help practitioners of age-period-cohort (APC) methods articulate their analytic assumptions and validate their results. The guidelines are applied to APC analyses results recently published in American Journal of Epidemiology about black-white differences in U.S. heart disease mortality. From these analyses, the paper concludes that some APC methods produce inconsistent results that are highly sensitive to researcher manipulation, while results estimated from other APC methods are robust to researcher manipulation and consistent across APC models.

Overall, the paper is reasonably well written, the analyses are clearly described (with data that can be used for independent verification), and the conclusions are well taken therefrom.

Here are a couple of things to attend to in a revision:

In the Data section, you state: "From the data in Table 1, 18 10-year birth cohorts can be calculated as linear outcomes of Period-Age = Cohort." First, do you mean 5-year birth cohorts, as the data in Table 1 are in 5-year age and time period groupings? Second, it would be good to indicate how the number of cohorts is calculated from the table.

It might be meritorious to include a brief statement on the concept of estimable functions in CGLIM APC models, how this has played a key role in the APC body of statistical methodology literature (going back to Kupper et al. 1985), how a statistical test (in Yang et al. 2008 and Yang and Land 2013) can be applied to CGLIM estimates (such as those in your various figures) of the A, P, and C coefficients to assess the extent to which they are within sampling error of the Intrinsic Estimator (which does satisfy the Kupper et al. condition).

Minor comment: Check the References for completeness and consistency of citations thereof in the text.

Reviewer #2: The history of the cottage industry of APC analysis has been full of contentious debates over the validities of various methodological approaches to estimating the APC regression models and further identifying the meaningful or “true” temporal trends attributable to period or cohort effects. While there is consensus that the APC analysis is theoretically and substantively important and serves a major tool for the surveillance of population level change related to time critical for public health and other social, political, and economic problems, the area of APC analysis has been plagued by confusions as a result of lack of understandings of the nature of the “model identification problem”, wrongful criticisms of existing and new methods arising from such misunderstandings, and consequently inadequate applications of analytic approaches (descriptive and multivariate). Suffice it to say, it is a such mess. The golden standard of good science, namely, replications of findings, has been elusive in this odd case. A practical guideline for how to adequately apply the best tools for a given topic and dataset and how to adjudicate among existing solutions is sorely needed.

This paper does just that! I have seen a handful of papers that have attempted at this goal, but none has done as well and thorough a job as this one. The problem with prior studies trying to defeat certain methodologies or recommending new ones is that they are charged with priori conclusions – they have decided what to reject or support prior to the actual algebraic development. A typical practice is to show through simulations and numerical applications that the method works or does not work without mathematical proofs. Simulations can be arbitrary and tailored to fit the intended conclusion. It has never been acceptable in the field of statistics to be the complete procedure for establishing a new method. This paper has truly achieved the goal of objective validation which has largely been absent so far in this mine field. It does so by showing the effect of attending to both the internal and external validity and reliability. By providing concrete criteria for validity, it allows analysts to make conclusions with confidence as opposed to being subjected to judgements made by others. After two decades of research on this topic, I see its values in guiding practitioners in their work and I will most happily follow the recommended three steps in my future studies.

A small suggestion: p.6., 1.c the term “researcher involvement” is a bit vague and can be more clearly stated as something like “subjective choice” or “arbitrary constraint”.

Reviewer #3: The goal of this paper is to overcome the "more heat than light" problem resulting from the debate about APC methods in social and medical sciences. The authors offer some concrete suggestions for researchers who use these methods and illustrate the usefulness of these suggestions through a reanalysis of Kramer et al. 2015 by 8 different "imaginary" research teams. The authors find that neither Kramer and colleagues' view of the "APC toolbox" nor the skeptical view by Harper that a researcher can find anything she wants using different methods is supported. In their analysis, most of the methods converge on similar findings and only the CGLIMs (of the sort used by Kramer) diverge. The authors conclude that neither view is right and that by explicitly comparing different estimators one can get a better handle on trends in the data.

In general, I find that this is a valuable contribution. I like the idea of the hypothetical 8 research teams. I completely agree with the authors' suggestions 1 and 3.

I have a couple of fairly minor concerns about the paper. The first is that I don't this suggestion #2 is stricly necessary. In most cases, there is literally no difference between estimating (say) two separate models for men and women and estimating a model where all covariates are interacted with sex. I realize that for many models (like the HAPC-CCREM) doing this properly would involve creating separate period and cohort variable values for men and women so that the random intercepts are estimated separately. Many researchers probably would not do this (or think of this). So, as a practical matter, perhaps the authors' suggestion is reasonable. But it might be worth noting that there are alternatives.

The second (minor) concern is whether certain methods are more likely to agree with each other in general, making "votes" between approaches not quite independent. I am very familiar with HAPC-CCREMs and CGLIMs because I work in a field that almost always uses individual data rather than tables. So I'm less familiar with (say) the IE. But if methods have similar assumptions might they not tend to agree even if they are "wrong"? That may be worth mentioning.

In general, however, this paper is a very valuable practical contribution and solid advice. Researchers using any techniques should always check for robustness of their results to alternative reasonable specifications. This set of guidelines should help bring this into being in the APC world.

Reviewer #4: Comments for the authors

I do not find any technical problems with the paper, but I also don’t find anything new here. There have been a number of papers over the years that have offered suggestions for appropriate use of APC methods, including some of the papers cited by the authors. The suggestions discussed and demonstrated here have each been discussed in other papers. It’s not clear that this paper makes a significant contribution to the literature. I would add that I don’t find APC analyses in general to be particularly useful, because they don’t answer a theory-driven question. Instead, one is left without an explanation for exactly what produced period or cohort effects. It is more advisable, rather than adopting yet another set of standards for conducting these atheoretical analyses, to simply include the cohort or period factors into the model that produce the effects. That approach doesn’t require arbitrary decisions to break linear dependence of effects.

**********

6. PLOS authors have the option to publish the peer review history of their article (what does this mean?). If published, this will include your full peer review and any attached files.

If you choose “no”, your identity will remain anonymous but your review may still be made public.

**Do you want your identity to be public for this peer review?** For information about this choice, including consent withdrawal, please see our Privacy Policy.

Reviewer #1: No

Reviewer #2: No

Reviewer #3: No

Reviewer #4: No

[NOTE: If reviewer comments were submitted as an attachment file, they will be attached to this email and accessible via the submission site. Please log into your account, locate the manuscript record, and check for the action link "View Attachments". If this link does not appear, there are no attachment files.]

While revising your submission, please upload your figure files to the Preflight Analysis and Conversion Engine (PACE) digital diagnostic tool, https://pacev2.apexcovantage.com/. PACE helps ensure that figures meet PLOS requirements. To use PACE, you must first register as a user. Registration is free. Then, login and navigate to the UPLOAD tab, where you will find detailed instructions on how to use the tool. If you encounter any issues or have any questions when using PACE, please email PLOS at figures@plos.org. Please note that Supporting Information files do not need this step.

# Author comment: Author response to Decision Letter 0

**DOI:** 10.1371/journal.pone.0238871.r002

30 Jul 2020

Please see "Response to Reviewers" file attached.

Attachment Submitted filename: Response to Review.docx Click here for additional data file.

# Peer review: Decision Letter 1

**DOI:** 10.1371/journal.pone.0238871.r003

**Authors:** Bryan L. Sykes

26 Aug 2020

Clarifying Assumptions in Age-Period-Cohort Analyses and Validating Results

PONE-D-20-06144R1

Dear Dr. Masters,

We’re pleased to inform you that your manuscript has been judged scientifically suitable for publication and will be formally accepted for publication once it meets all outstanding technical requirements.

Within one week, you’ll receive an e-mail detailing the required amendments. When these have been addressed, you’ll receive a formal acceptance letter and your manuscript will be scheduled for publication.

An invoice for payment will follow shortly after the formal acceptance. To ensure an efficient process, please log into Editorial Manager at http://www.editorialmanager.com/pone/, click the 'Update My Information' link at the top of the page, and double check that your user information is up-to-date. If you have any billing related questions, please contact our Author Billing department directly at authorbilling@plos.org.

If your institution or institutions have a press office, please notify them about your upcoming paper to help maximize its impact. If they’ll be preparing press materials, please inform our press team as soon as possible -- no later than 48 hours after receiving the formal acceptance. Your manuscript will remain under strict press embargo until 2 pm Eastern Time on the date of publication. For more information, please contact onepress@plos.org.

Kind regards,

Bryan L. Sykes, Ph.D.

Academic Editor

PLOS ONE

Additional Editor Comments (optional):

Reviewers' comments:

Reviewer's Responses to Questions

**Comments to the Author**

1. If the authors have adequately addressed your comments raised in a previous round of review and you feel that this manuscript is now acceptable for publication, you may indicate that here to bypass the “Comments to the Author” section, enter your conflict of interest statement in the “Confidential to Editor” section, and submit your "Accept" recommendation.

Reviewer #1: All comments have been addressed

Reviewer #3: All comments have been addressed

**********

2. Is the manuscript technically sound, and do the data support the conclusions?

The manuscript must describe a technically sound piece of scientific research with data that supports the conclusions. Experiments must have been conducted rigorously, with appropriate controls, replication, and sample sizes. The conclusions must be drawn appropriately based on the data presented. 

Reviewer #1: Yes

Reviewer #3: Yes

**********

3. Has the statistical analysis been performed appropriately and rigorously? 

Reviewer #1: Yes

Reviewer #3: Yes

**********

4. Have the authors made all data underlying the findings in their manuscript fully available?

The PLOS Data policy requires authors to make all data underlying the findings described in their manuscript fully available without restriction, with rare exception (please refer to the Data Availability Statement in the manuscript PDF file). The data should be provided as part of the manuscript or its supporting information, or deposited to a public repository. For example, in addition to summary statistics, the data points behind means, medians and variance measures should be available. If there are restrictions on publicly sharing data—e.g. participant privacy or use of data from a third party—those must be specified.

Reviewer #1: (No Response)

Reviewer #3: Yes

**********

5. Is the manuscript presented in an intelligible fashion and written in standard English?

PLOS ONE does not copyedit accepted manuscripts, so the language in submitted articles must be clear, correct, and unambiguous. Any typographical or grammatical errors should be corrected at revision, so please note any specific errors here.

Reviewer #1: Yes

Reviewer #3: Yes

**********

6. Review Comments to the Author

Please use the space provided to explain your answers to the questions above. You may also include additional comments for the author, including concerns about dual publication, research ethics, or publication ethics. (Please upload your review as an attachment if it exceeds 20,000 characters)

Reviewer #1: The revisions to this manuscript and the responses to the previous review are adequate and the paper now is acceptable for publication.

Reviewer #3: (No Response)

**********

7. PLOS authors have the option to publish the peer review history of their article (what does this mean?). If published, this will include your full peer review and any attached files.

If you choose “no”, your identity will remain anonymous but your review may still be made public.

**Do you want your identity to be public for this peer review?** For information about this choice, including consent withdrawal, please see our Privacy Policy.

Reviewer #1: No

Reviewer #3: No

# Editor report: Acceptance letter

**DOI:** 10.1371/journal.pone.0238871.r004

**Authors:** Bryan L. Sykes

17 Sep 2020

PONE-D-20-06144R1 

Clarifying Assumptions in Age-Period-Cohort Analyses and Validating Results 

Dear Dr. Masters:

I'm pleased to inform you that your manuscript has been deemed suitable for publication in PLOS ONE. Congratulations! Your manuscript is now with our production department. 

If your institution or institutions have a press office, please let them know about your upcoming paper now to help maximize its impact. If they'll be preparing press materials, please inform our press team within the next 48 hours. Your manuscript will remain under strict press embargo until 2 pm Eastern Time on the date of publication. For more information please contact onepress@plos.org.

If we can help with anything else, please email us at plosone@plos.org. 

Thank you for submitting your work to PLOS ONE and supporting open access. 

Kind regards, 

PLOS ONE Editorial Office Staff

on behalf of

Dr. Bryan L. Sykes 

Academic Editor

PLOS ONE
